# Supplementary material for: A macrophage-based screen identifies antibacterial compounds selective for intracellular Salmonella Typhimurium
Source: Nat Commun. 2019 Jan 14;10:197. doi: 10.1038/s41467-018-08190-x (PMC6331611; doi:10.1038/s41467-018-08190-x)
Supplement: Supplementary file 1 — Supplementary Information [file 41467_2018_8190_MOESM1_ESM.pdf]

SUPPLEMENTARY INFORMATION

A macrophage-based screen identifies antibacterial compounds selective for intracellular *Salmonella* Typhimurium

Ellis et al.

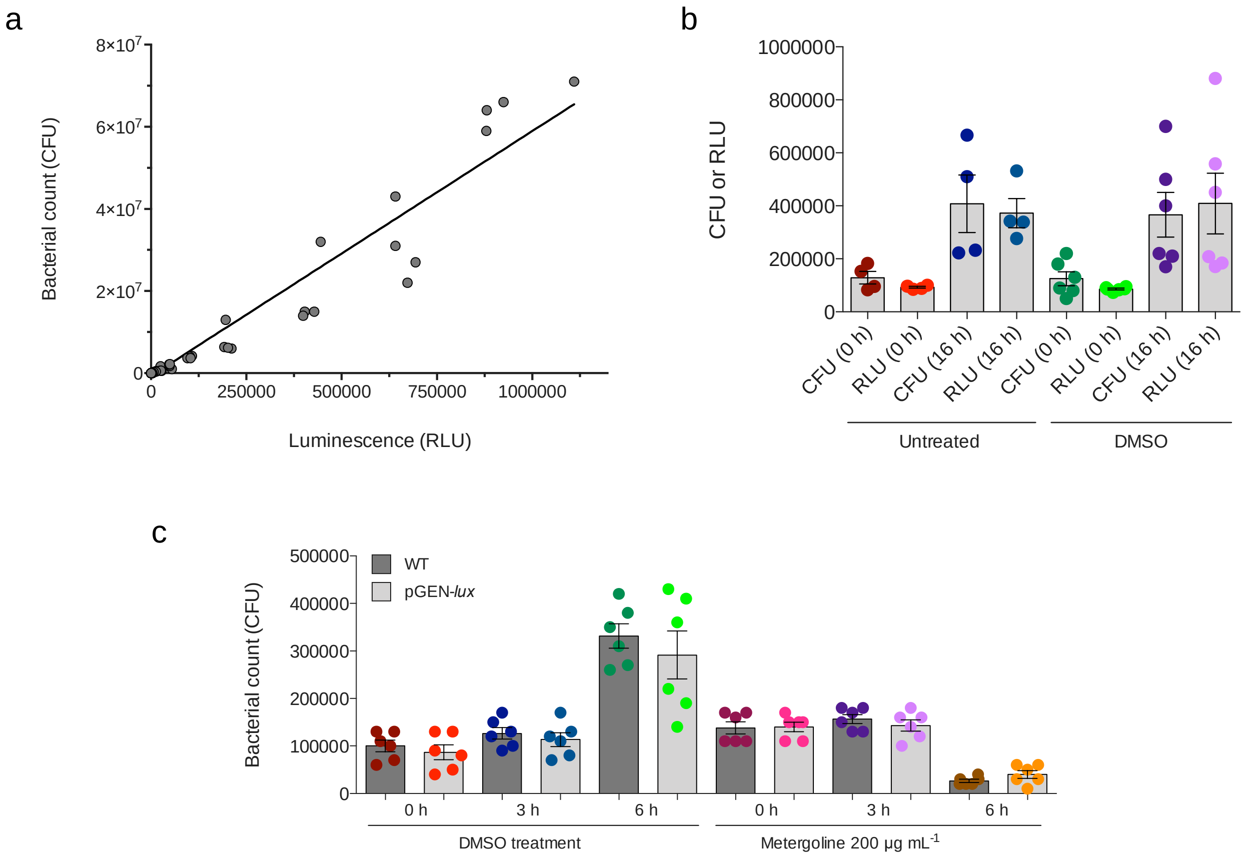

Supplementary Figure 1. Optimization of intracellular screening workflow. **a** Colony forming unit (CFU) to luminescence correlation from *S.* Tm transformed with the pGEN plasmid expressing constitutive luciferase from the *em7* promoter (pGEN-*lux*). **b** Luminescence (relative light units, RLU) and CFU of *S.* Tm transformed with pGEN-*lux*, internalized in untreated (left) or DMSO-treated (right) RAW264.7 macrophages, measured directly after gentamicin treatment to kill extracellular bacteria (0 h) and 16 h later. Bar plots depict the mean of 4-6 biological replicates, error bars indicate s.e.m. **c** *S.* Tm (dark grey) and *S.* Tm transformed with pGEN-*lux* (light grey) were internalized into RAW264.7 macrophages and treated with DMSO (left) or 200 µg mL^{-1} metergoline (right). Macrophages were lysed and CFU were enumerated immediately after gentamicin treatment (0 h), at 3 h post-infection, and at 6 h post-infection. Bar plots depict the mean of 6 biological replicates, error bars indicate s.e.m.

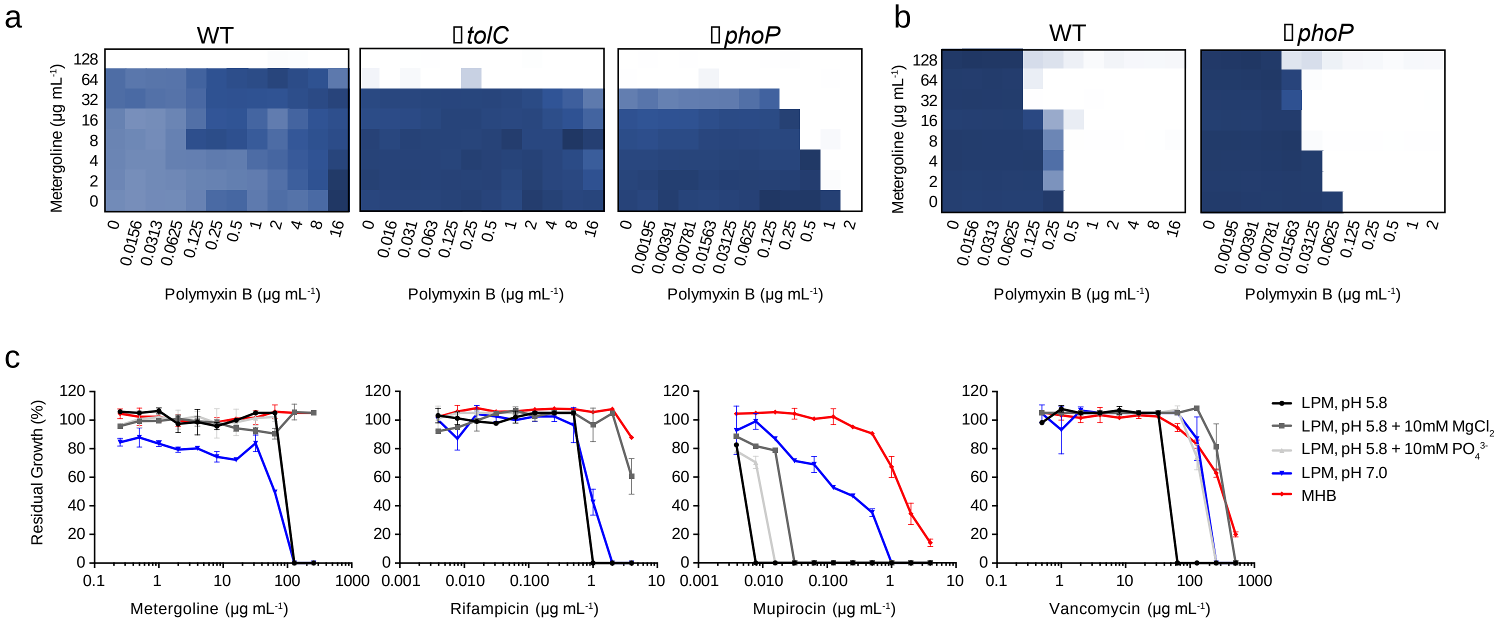

Supplementary Figure 2. Impact of LPM or mutants on metergoline activity. **a** Chequerboard broth microdilution assay measuring synergy between metergoline and polymyxin B in LPM. *S.* Tm WT, Δ*tolC*, or Δ*phoP* were grown in LPM with the indicated concentrations of polymyxin B and metergoline. Darker colour indicates higher cell density. **b** Chequerboard broth microdilution assay measuring synergy between metergoline and polymyxin B in MHB. In **a** and **b**, data shown are representative of at least two experiments. **C** Potency analysis of metergoline, rifampicin, mupirocin, or vancomycin in MHB or variants of LPM. LPM was adjusted to pH 7.0 or supplemented with 10 mM $MgCl_2$ or 10 mM PO_4^{3-} as indicated. Residual growth was calculated as a percentage relative to an untreated control in each media. Points and error bars show the mean and s.e.m. from two biological replicates.

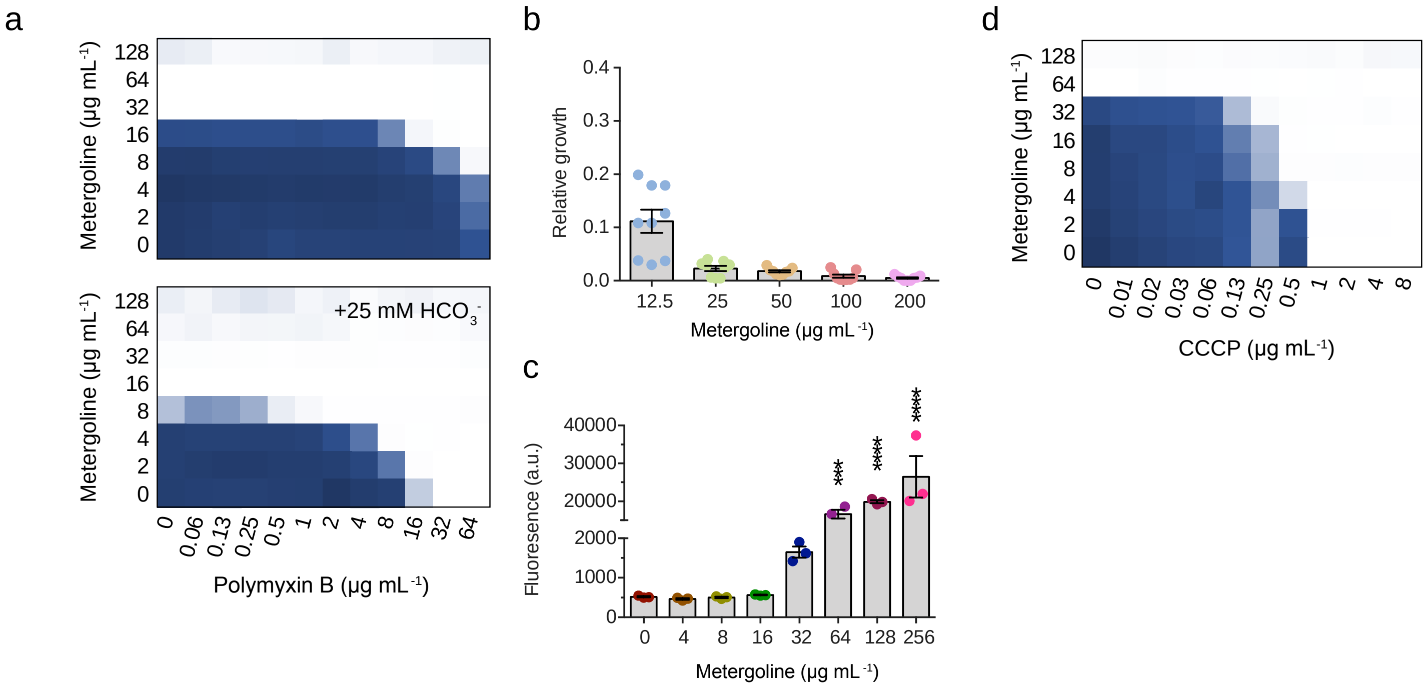

Supplementary Figure 3. Activity of metergoline against methicillin-resistant *Staphylococcus aureus* (MRSA). **a** Chequerboard broth microdilution assay showing synergy between polymyxin B and metergoline against MRSA grown in MHB, or MHB supplemented with 25 mM bicarbonate (HCO_3^-). **b** Intracellular replication of MRSA measured in bone-marrow derived macrophages isolated from C57BL/6 mice. Relative growth reflects replication over 4 h, normalized to bacterial growth in macrophages treated with DMSO. Metergoline was added at the indicated concentrations. Bar plots depict the mean of 9 biological replicates, error bars indicate s.e.m. **c** The effect of metergoline on cytoplasmic membrane potential was measured by the $DiSC_3(5)$ assay, following a 1 min exposure to the indicated concentrations of metergoline. Bar plots depict the mean fluorescence of three biological replicates, error bars indicate s.e.m. All groups were compared to 0 µg mL^{-1} metergoline via one-way ANOVA with Holm-Sidak's multiple test correction. ***P<0.001, ****P<0.0001. **d** Chequerboard analysis showing synergy between metergoline and CCCP against MRSA grown in MHB. In **a** and **b** darker colour indicates higher bacterial growth and data are representative of two experiments.

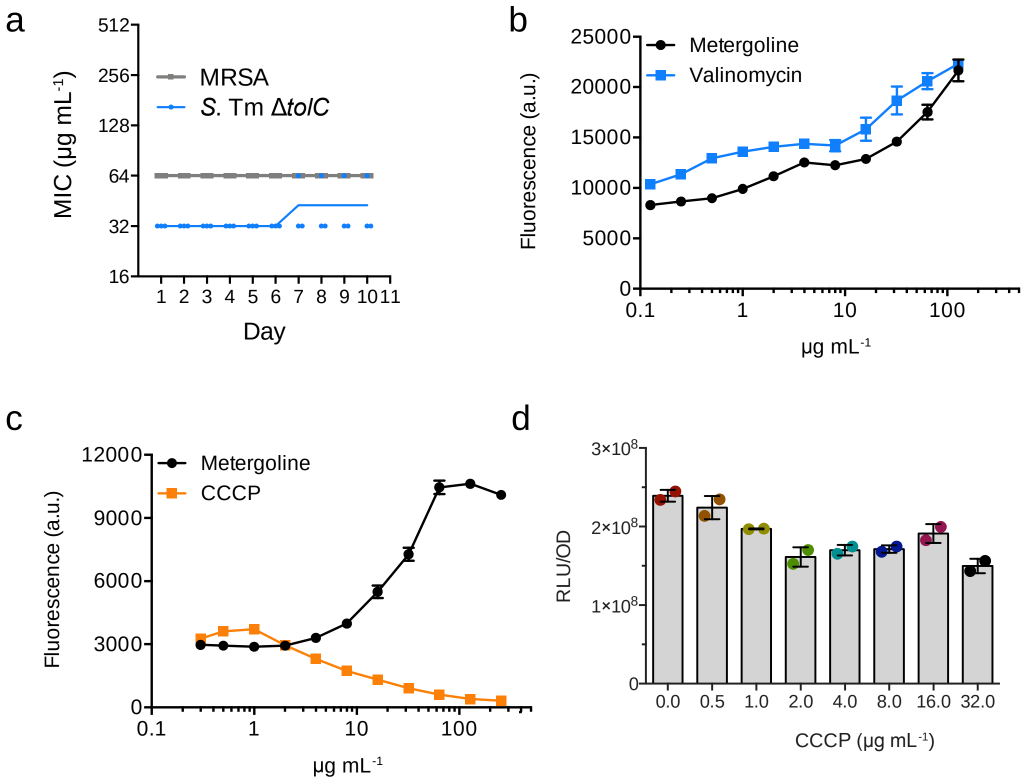

Supplementary Figure 4. Serial-passage experiment with metergoline and control experiments for DiSC$_3$(5) and ATP assays. **a** The MIC of metergoline against three independent lineages of MRSA or Δ*tolC S.* Tm was measured daily for 10 d (see Methods). Cells from the highest concentration of metergoline with detectable growth were used each day to inoculate MIC plates. Points show the MIC for individual lineages and the connected line shows the mean MIC. **b-c** Effect of metergoline, valinonmycin (**b**), and CCCP (**c**) on inner membrane potential was measured by the DiSC$_3$(5) assay. WT *S.* Tm was grown in MHB with 10 mM EDTA to an OD_{600} ~ 1. Cells were loaded with DiSC$_3$(5) prior to 1 min incubation with compound. Note that 100 mM KCl was added to cells in **b** as valinomycin in K^+-dependent. Error bars show s.e.m. for technical triplicates. **d** WT *S*. Tm was grown in MHB with 1 mM EDTA to OD_{600} = 0.2 and then grown in the presence of CCCP for 30 min at 37ºC. Cellular ATP levels were estimated by luciferase activity (relative light units, RLU) normalized to optical density (OD_{600}). Bar plots depict the mean of two biological replicates, error bars indicate s.d.

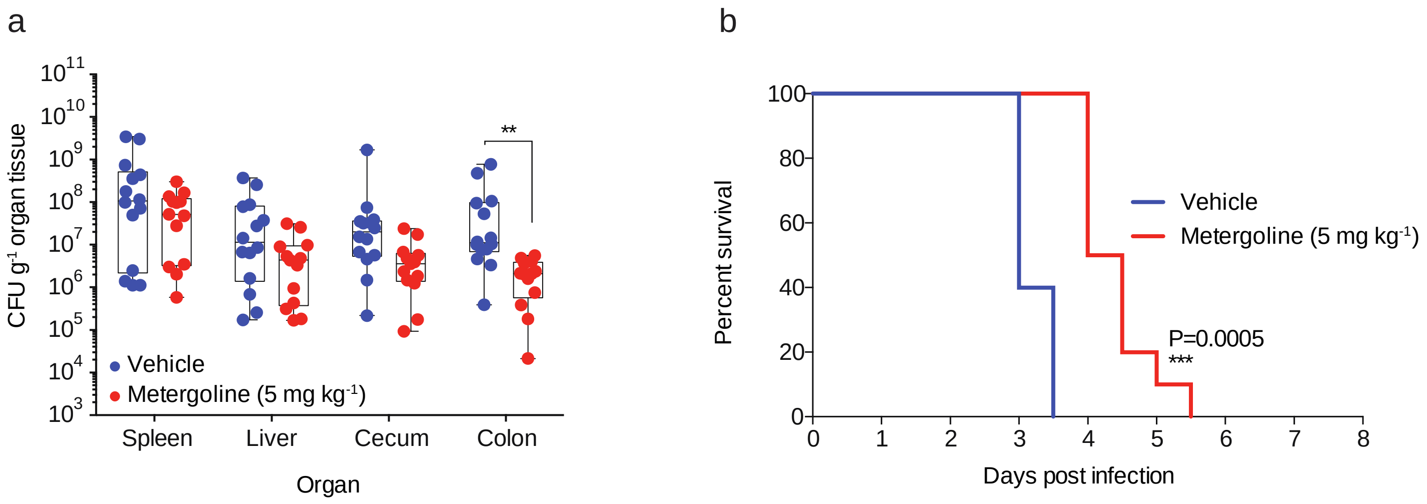

Supplementary Figure 5. *In vivo* efficacy of metergoline following delayed treatment administration. **a** Groups of mice were treated twice daily (every 12 h) with metergoline (5 mg kg^{-1}, red), or DMSO (5% in DMEM, blue) by i.p. injection. Treatments were administered beginning 12 h after initial infection. Mice were euthanized at experimental endpoint (60 h post-infection). Bacterial load in the spleen, liver, cecum, and colon was determined by selective plating in the presence of 150 µg mL^{-1} streptomycin. Data shown are from three separate experiments (n=5 per group). Box plot whiskers show the minimum to maximum values per group, lines in box plots show the median of each group. Groups were analyzed with a two-way ANOVA and corrected for multiple comparisons with a Holm-Sidak test. **b** For survival experiments, groups of mice (n=10) were treated twice daily (every 12 h) with metergoline (5 mg kg^{-1}, red), or DMSO (5% in DMEM, blue) by i.p. injection, and were euthanized at clinical endpoint. Treatments were administered beginning 12 h after initial infection. Survival curves shown are from two independent experiments. Groups were analyzed with a Gehan-Breslow-Wilcoxon test for survival curve differences. *P<0.05, **P<0.01, ***P<0.001, ****P<0.0001.

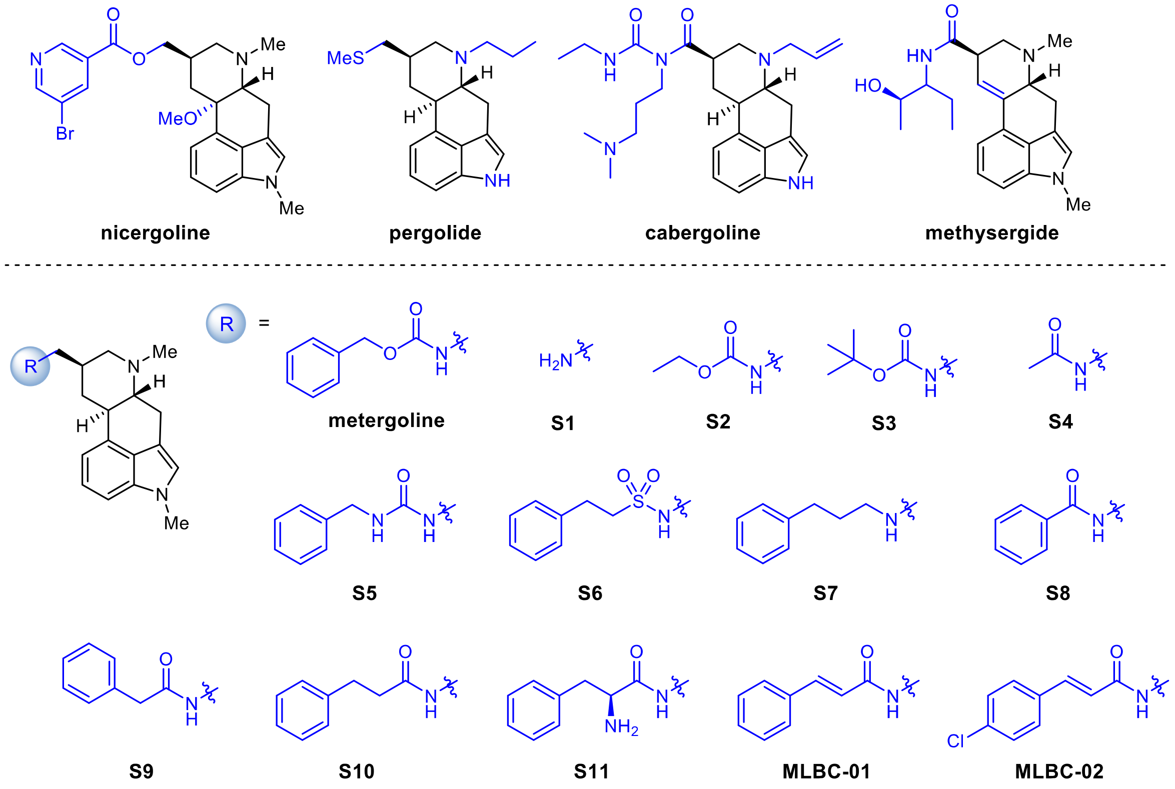

Supplementary Figure 6 Structure of metergoline analogues. Structural features differing from metergoline are indicated in blue.

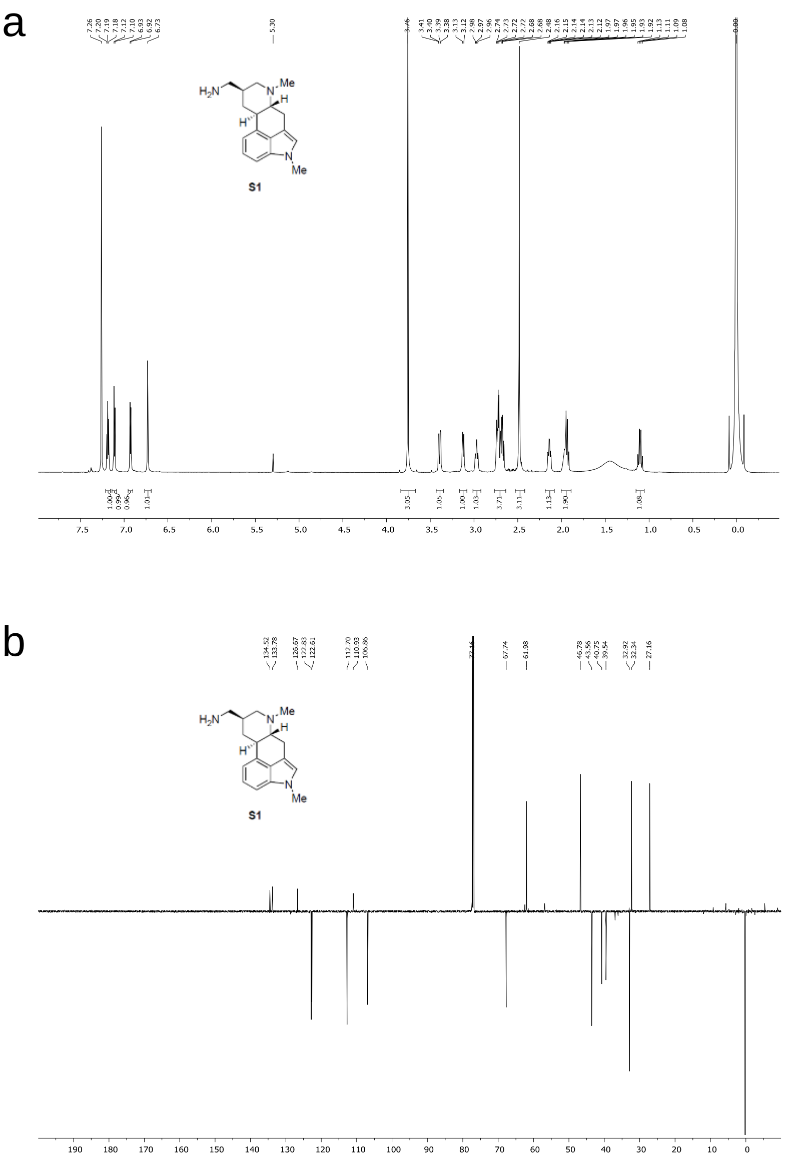

Supplementary Figure 7. NMR spectra for aminomethyl ergoline (**S1**). **a** ^{1}H NMR, 700 MHz, $CDCl_3$. **b** ^{13}C NMR, 175 MHz, $CDCl_3$. The structure of compound **S1** is shown.

a

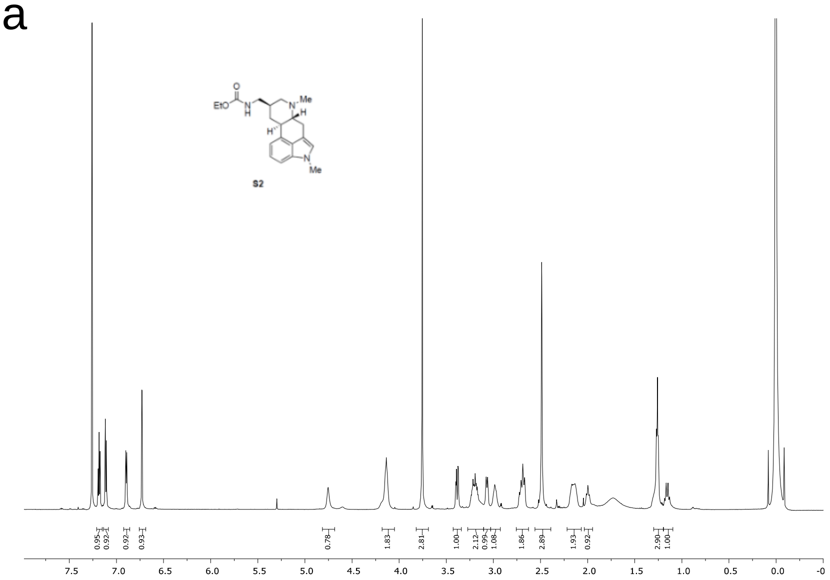

b

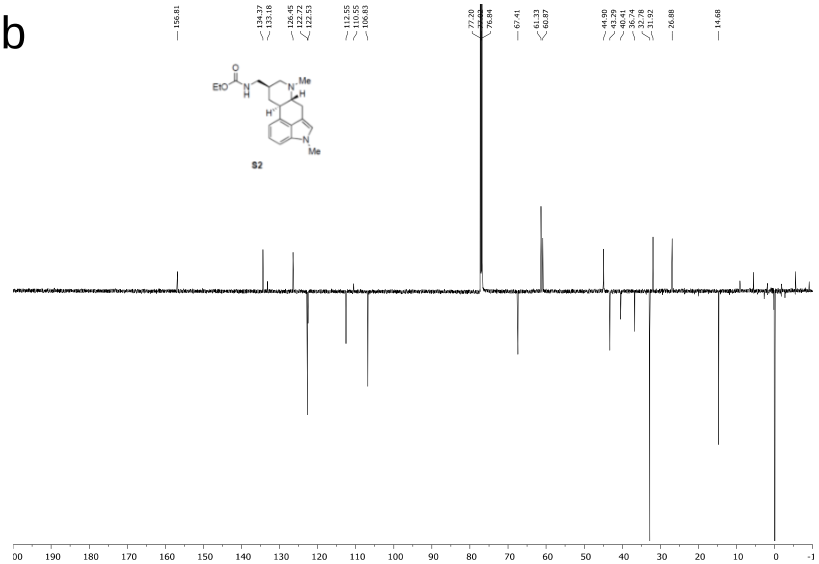

Supplementary Figure 8. NMR spectra for ethyl carbamate derivative (**S2**). **a** ^{1}H NMR, 700 MHz, $CDCl_3$. **b** ^{13}C NMR, 175 MHz, $CDCl_3$. The structure of compound **S2** is shown.

a

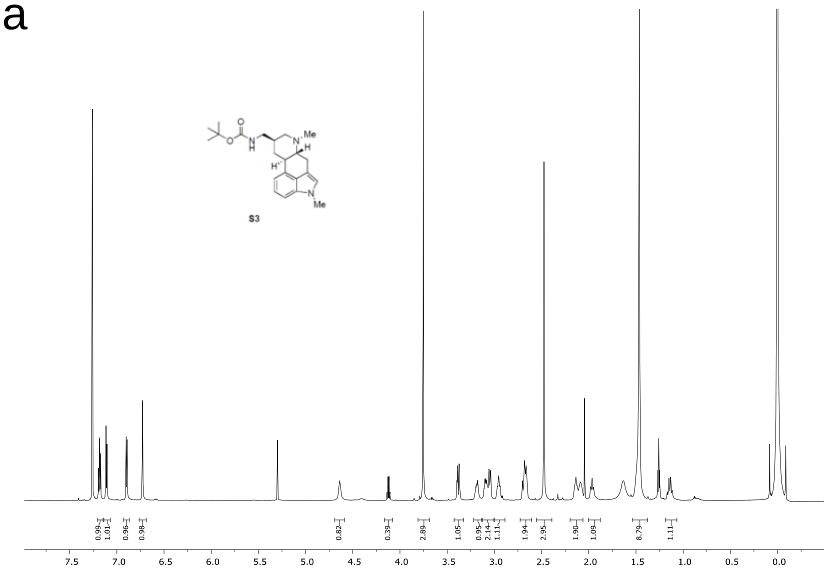

b

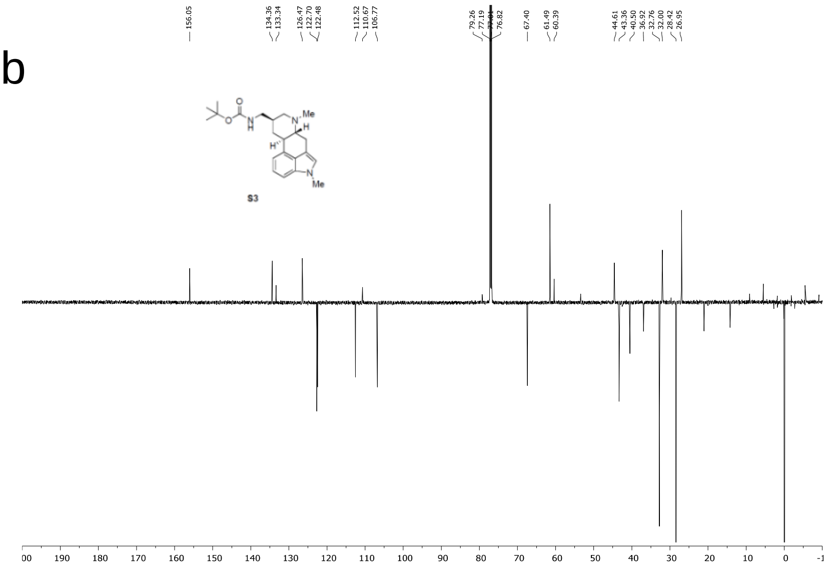

Supplementary Figure 9. NMR spectra for *N*-Boc ergoline derivative (**S3**). **a** ^{1}H NMR, 700 MHz, $CDCl_3$. **b** ^{13}C NMR, 175 MHz, $CDCl_3$. The structure of compound **S3** is shown.

a

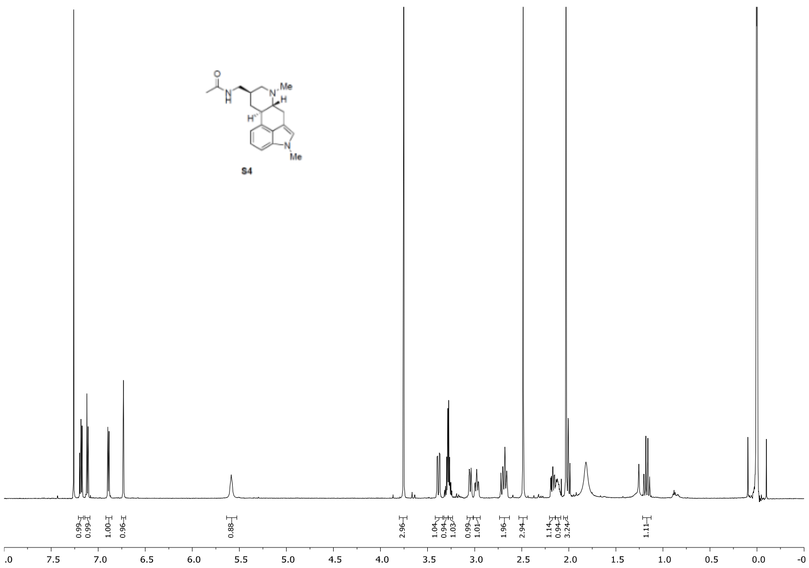

b

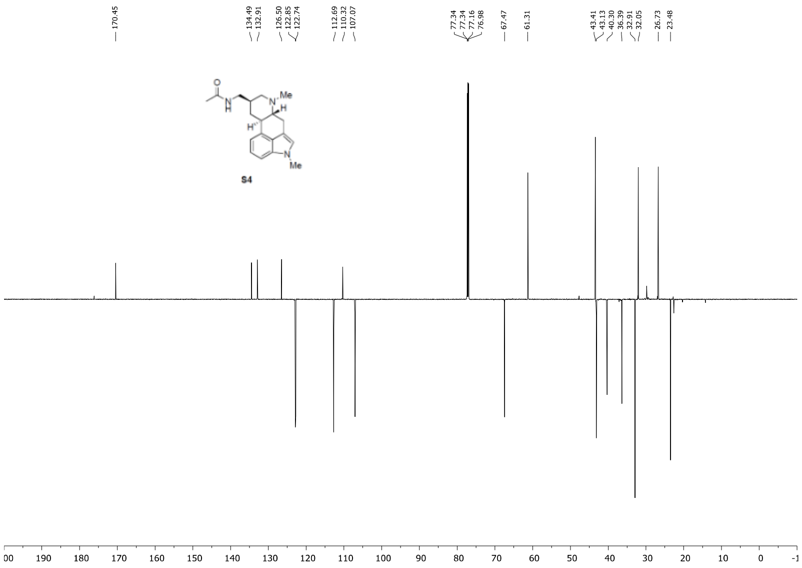

Supplementary Figure 10. NMR spectra for *N*-Acetyl ergoline derivative (**S4**). **a** ^{1}H NMR, 600 MHz, $CDCl_3$. **b** ^{13}C NMR, 175 MHz, $CDCl_3$. The structure of compound **S4** is shown.

a

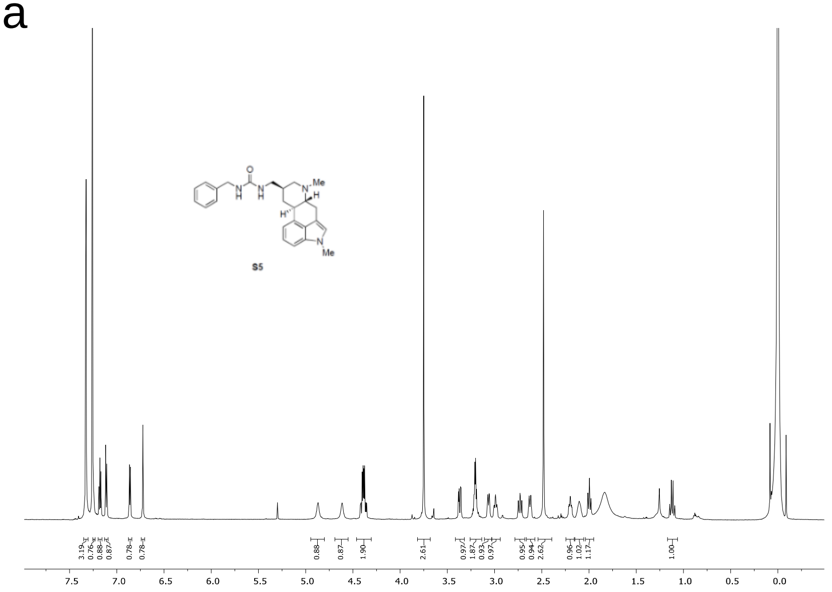

b

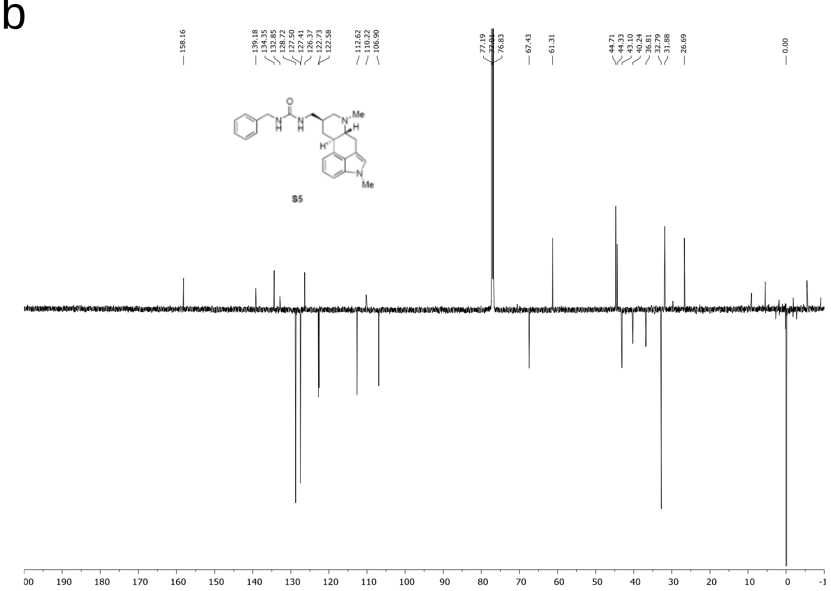

Supplementary Figure 11. NMR spectra for *N*-(*N*'-Benzyl) urea derivative (**S5**). **a** ^{1}H NMR, 700 MHz, $CDCl_3$. **b** ^{13}C NMR, 175 MHz, $CDCl_3$. The structure of compound **S5** is shown.

a

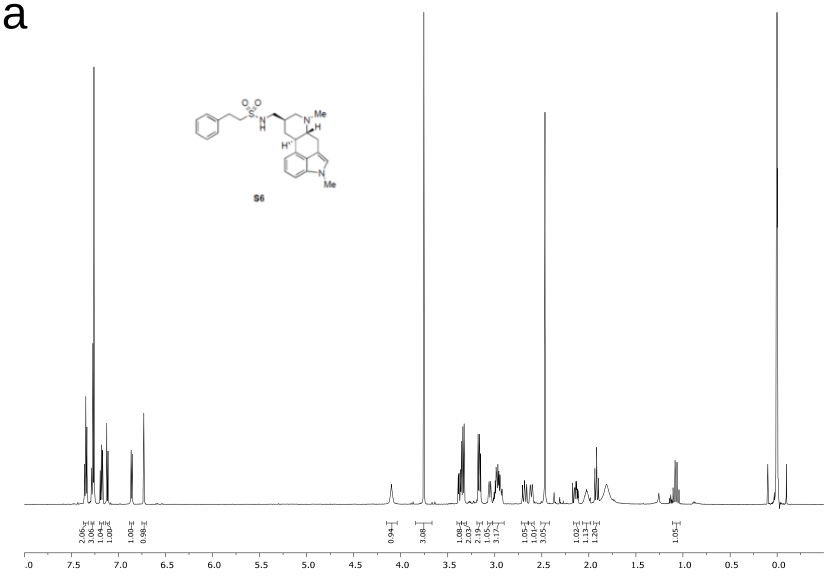

b

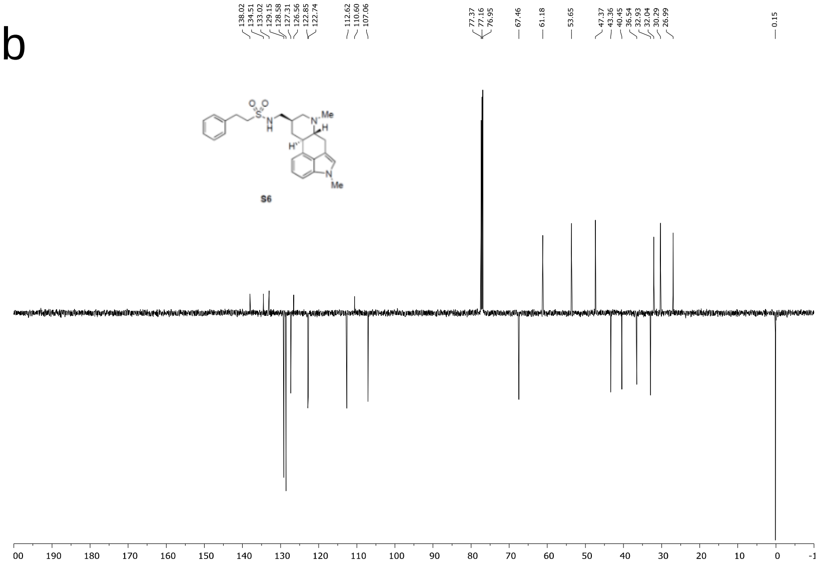

Supplementary Figure 12. NMR spectra for *N*-(2-Phenylethylsulfonyl) ergoline derivative (**S6**). **a** ^{1}H NMR, 600 MHz, $CDCl_3$. **b** ^{13}C NMR, 150 MHz, $CDCl_3$. The structure of compound **S6** is shown.

a

b

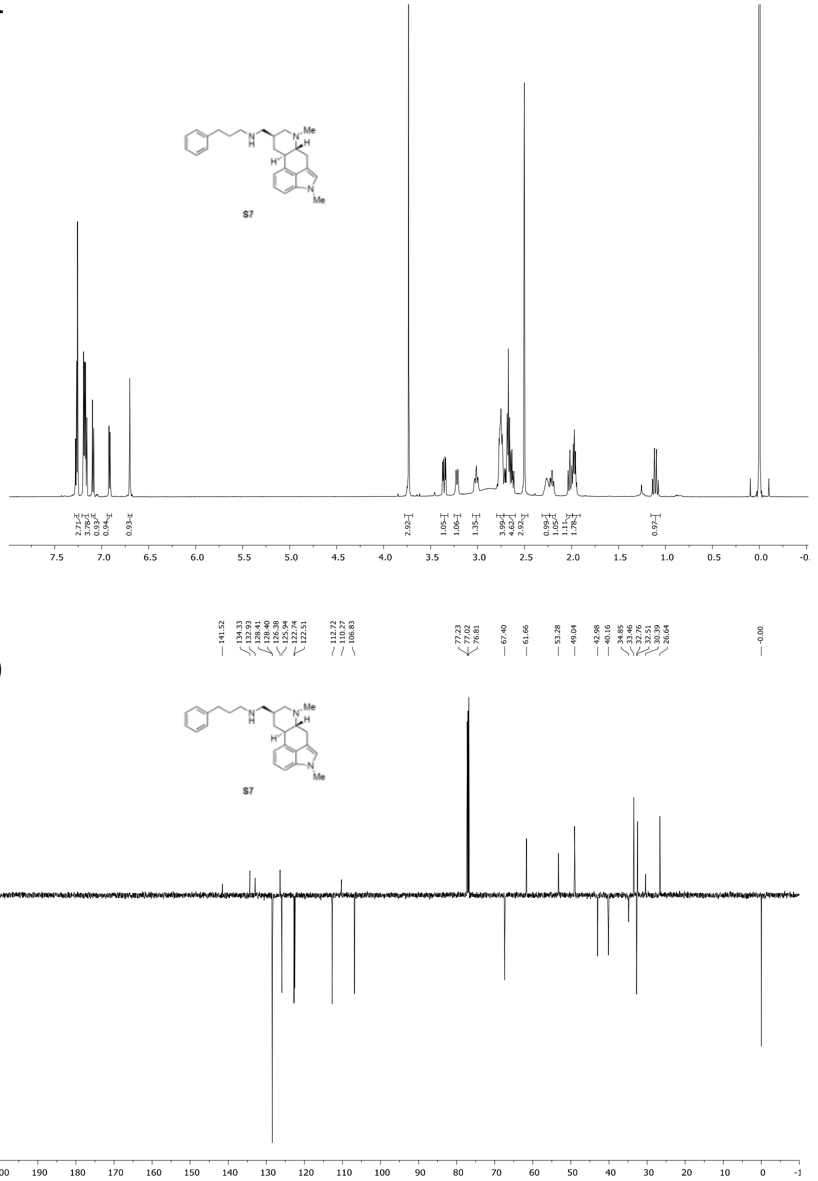

Supplementary Figure 13. NMR spectra for *N*-(3-Phenylpropyl) ergoline derivative (**S7**). **a** ^{1}H NMR, 600 MHz, $CDCl_3$. **b** ^{13}C NMR, 150 MHz, $CDCl_3$. The structure of compound **S7** is shown.

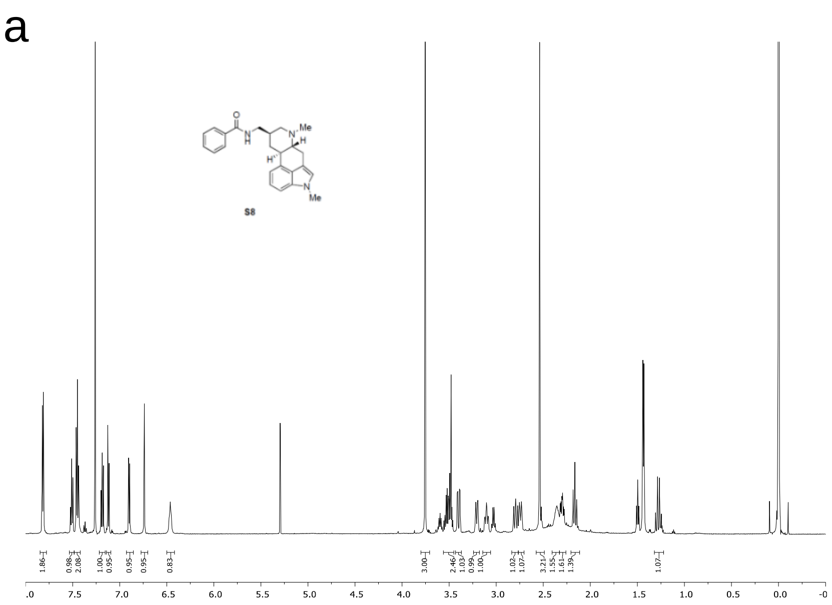

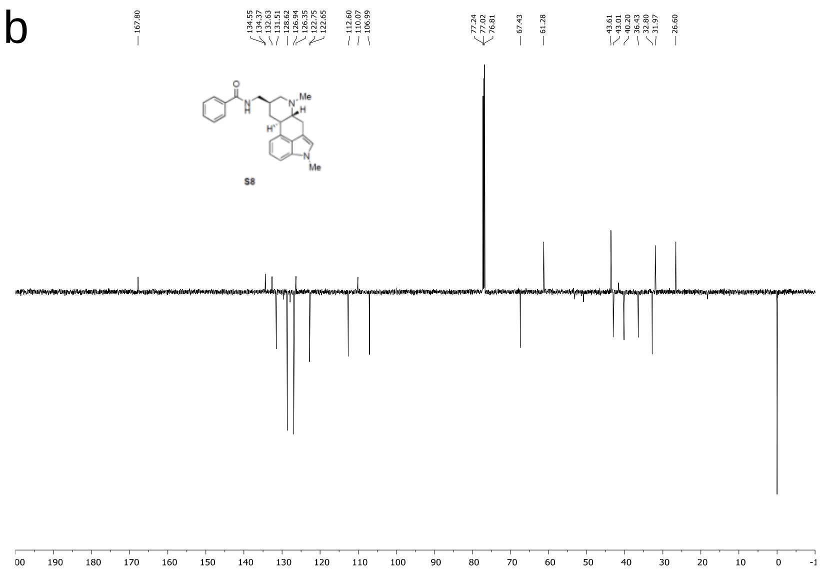

Supplementary Figure 14. NMR spectra for *N*-Benzoyl ergoline derivative (**S8**). **a** ^{1}H NMR, 600 MHz, $CDCl_3$. **b** ^{13}C NMR, 150 MHz, $CDCl_3$. The structure of compound **S8** is shown.

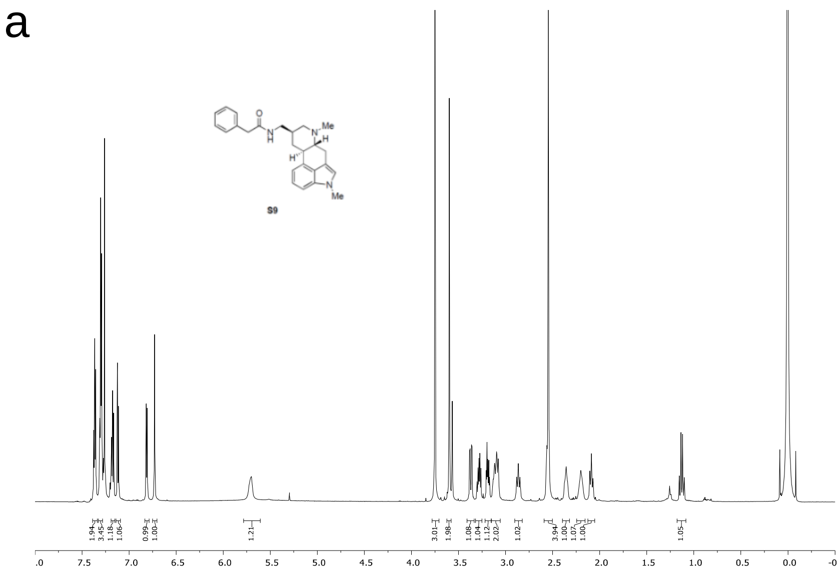

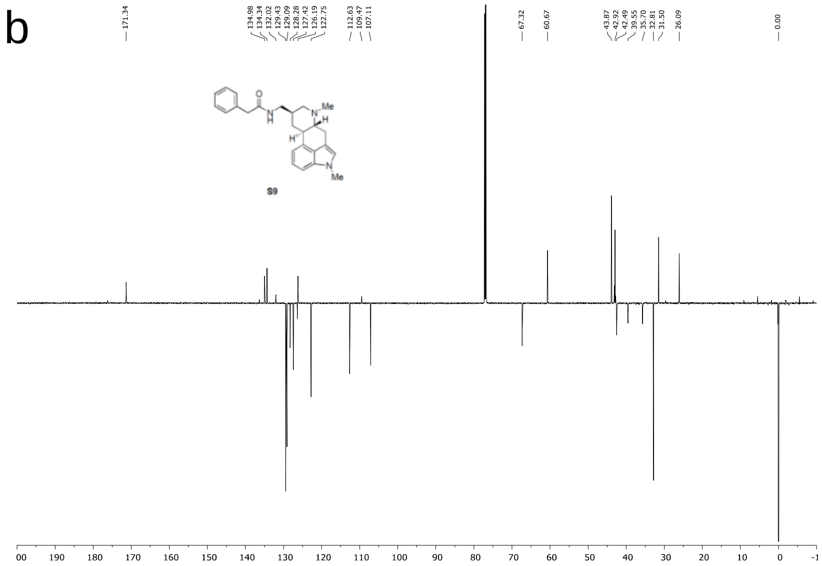

Supplementary Figure 15. NMR spectra for *N*-Phenylacetyl ergoline derivative (**S9**). **a** ^{1}H NMR, 700 MHz, $CDCl_3$. **b** ^{13}C NMR, 175 MHz, $CDCl_3$. The structure of compound **S9** is shown.

a

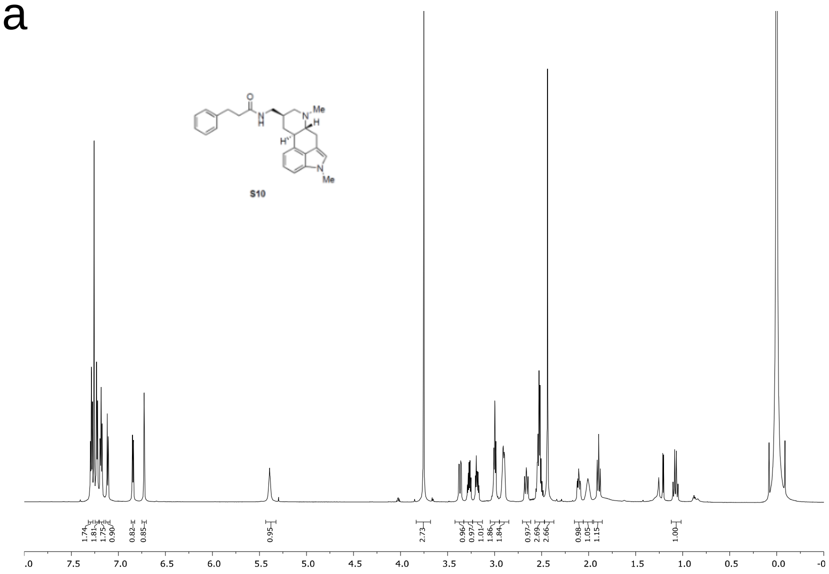

b

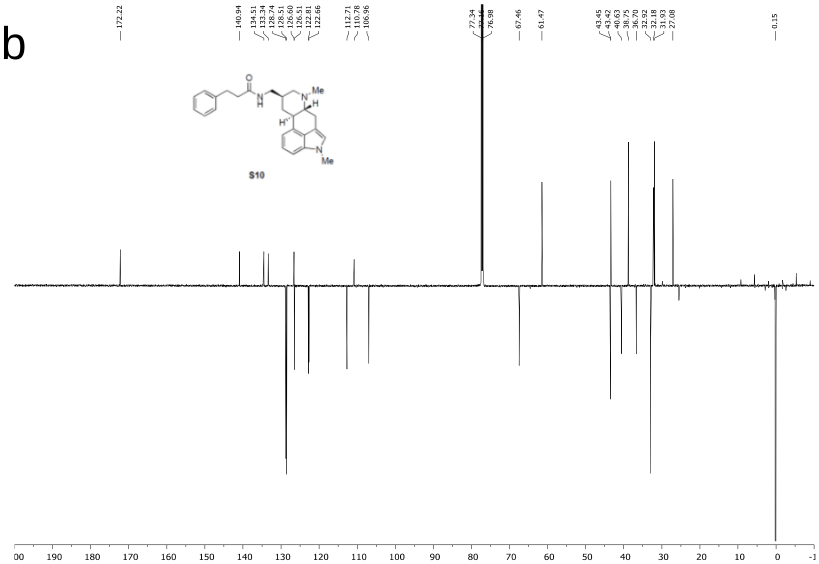

Supplementary Figure 16. NMR spectra for *N-(3*-Phenylpropanoyl) ergoline derivative (**S10**). **a** ^{1}H NMR, 700 MHz, $CDCl_3$. **b** ^{13}C NMR, 175 MHz, $CDCl_3$. The structure of compound **S10** is shown.

a

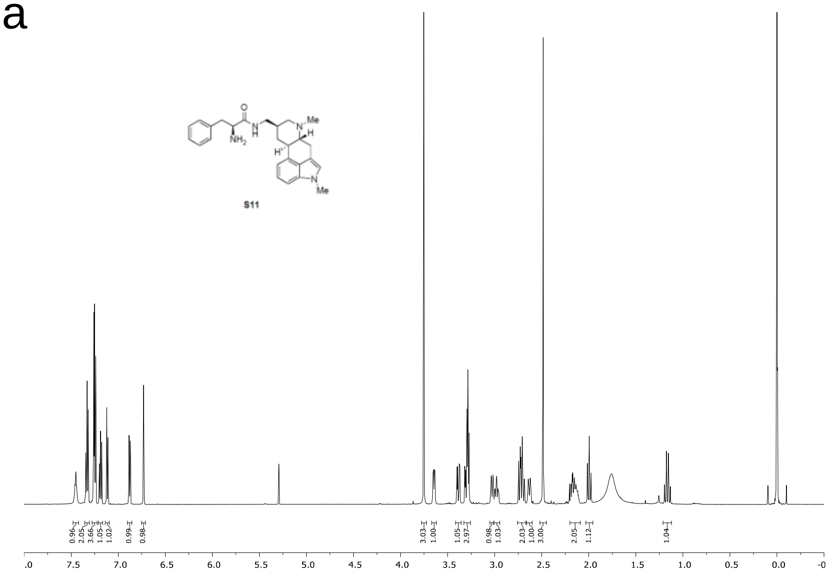

b

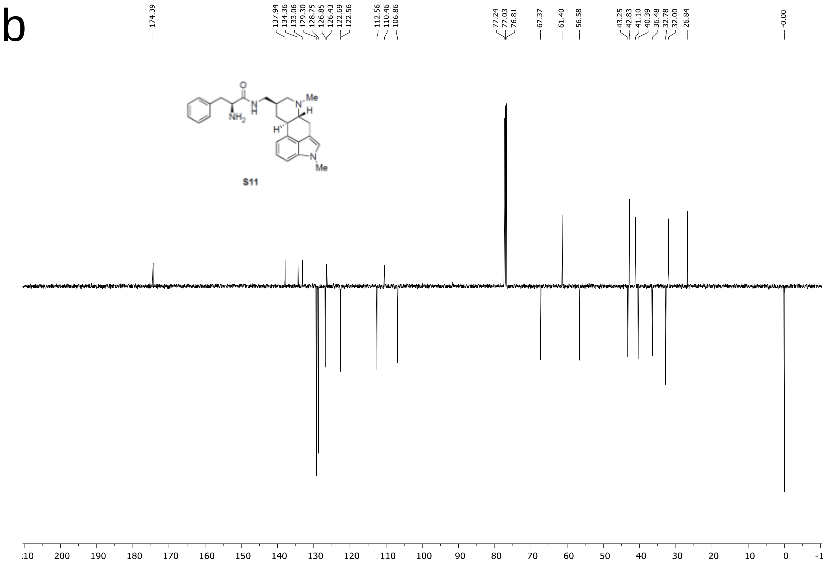

Supplementary Figure 17. NMR spectra for *N*-L-Phenylalanyl ergoline derivative (**S11**). **a** ^{1}H NMR, 600 MHz, $CDCl_3$. **b** ^{13}C NMR, 150 MHz, $CDCl_3$. The structure of compound **S11** is shown.

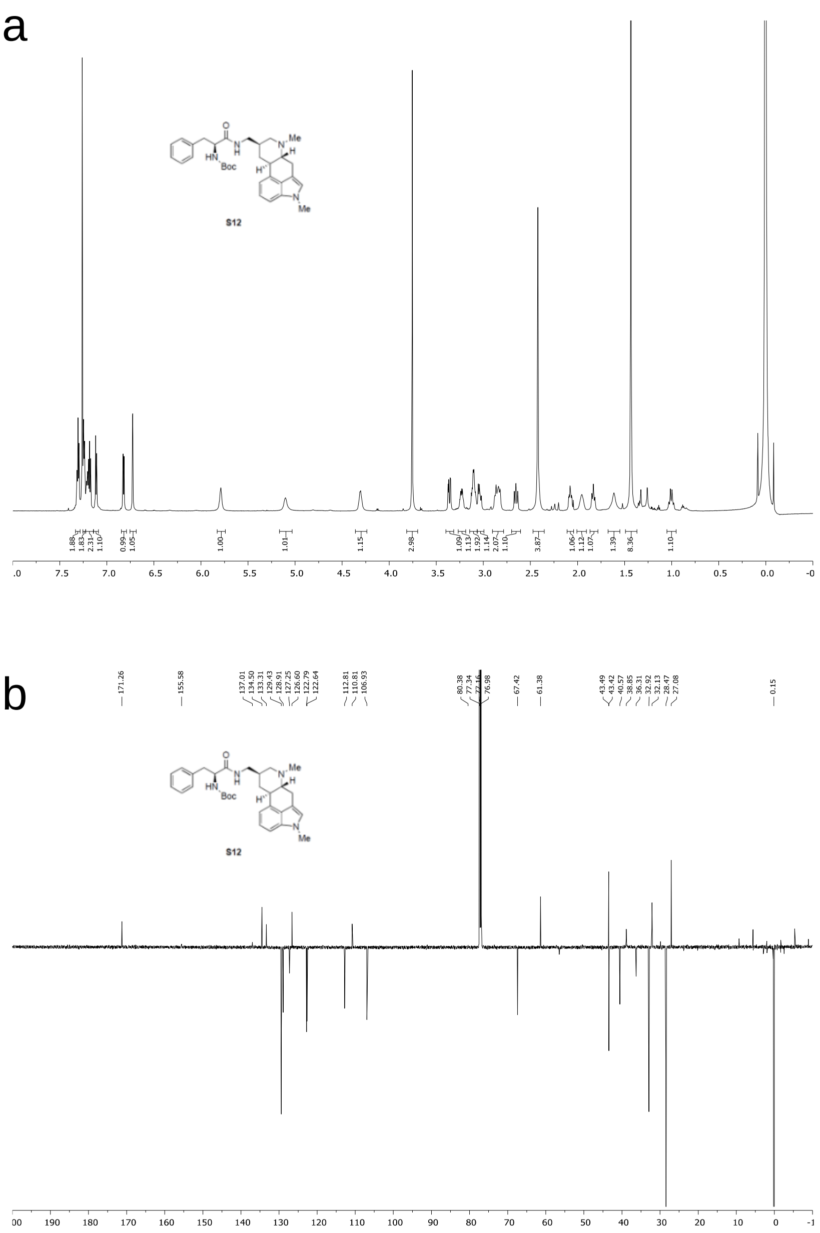

Supplementary Figure 18. NMR spectra for *N*-(*N*-Boc-L-Phenylalanyl) ergoline derivative (**S12**). **a** ^{1}H NMR, 700 MHz, $CDCl_3$. **b** ^{13}C NMR, 175 MHz, $CDCl_3$. The structure of compound **S12** is shown.

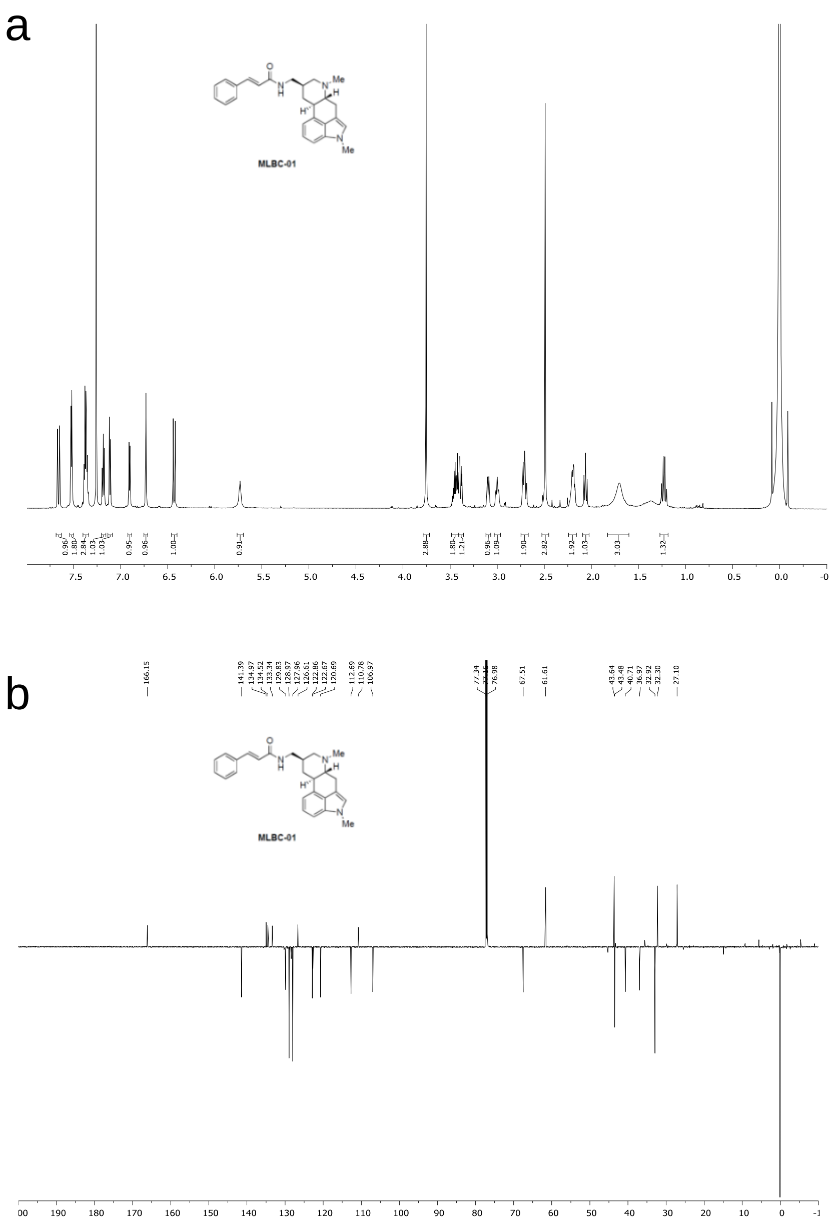

Supplementary Figure 19. NMR spectra for *N*-trans-Cinnamoyl derivative (**MLBC-01**). **a** ^{1}H NMR, 700 MHz, $CDCl_3$. **b** ^{13}C NMR, 175 MHz, $CDCl_3$. The structure of compound **MLBC-01** is shown.

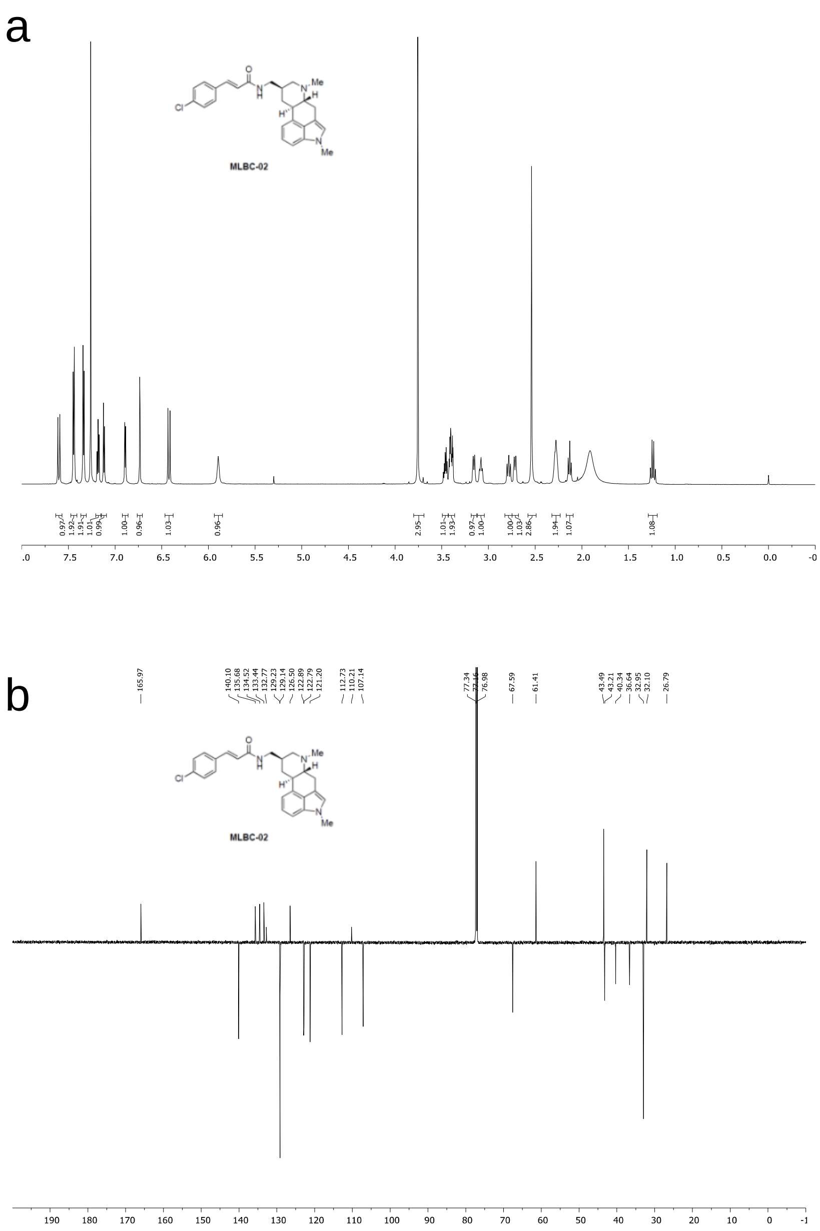

Supplementary Figure 20. NMR spectra for *N*-trans-Cinnamoyl derivative (**MLBC-02**). **a** ^{1}H NMR, 700 MHz, $CDCl_3$. **b** ^{13}C NMR, 175 MHz, $CDCl_3$. The structure of compound **MLBC-02** is shown.

Supplementary Table 1. Small molecule screening data

Category	Parameter	Description
Assay	Type of assay	Cell-based
	Target	Whole organism *Salmonella* Typhimurium SL1344
	Primary measurement	Measurement of optical density at 600 nm, luminescence
	Assay protocol	Methods section 'High-throughput compound screening'
Library	Library size	1600
	Library composition	Synthetic small molecules, off-patent FDA approved molecules, known bioactives
	Source	Microsource
Screen	Format	96-well plates (Corning), 3 replicates
	Concentration(s) tested	10 uM, 0.2% DMSO
	Plate controls	High controls: 0.1% DMSO<br>Low controls: 100 ug/mL rifampicin
	Reagent/compound dispensing system	Biomek FX liquid handler (Beckman Coulter Inc., Fullerton, CA)
	Detection instrument and software	Envision (Perkin Elmer, Waltham, MA)
	Assay validation/QC	For LPM screen: average (n=3) Z' score = 0.713, for RAW264.7 data see Figure S1
	Correction factors	Optical density readings were background corrected; Luminescence values were normalized to T_0
	Normalization	Plate and well effects normalized with interquartile mean
Post-HTS analysis	Hit criteria	Compounds reducing normalized growth more than 3σ below the
	Hit rate	LPM, 4.68%; RAW264.7, 6.44%
	Additional assay(s)	Dose-response and MIC determination *in vitro* and in macrophages
	Confirmation of hit purity and structure	Compounds repurchased and retested

Supplementary Table 2. Activity of metergoline and 17 structurally related compounds

	Minimum Inhibitory Concentration (μg mL^{-1})					RAW264.7	
	S. Tm WT			Δ*tolC*	MRSA	Growth[b]	Toxicity (%)[c]
Compound	MHB	EDTA[a]	LPM	MHB	MHB		
Metergoline	>128	64	128	32	32	0.08	2.9
nicergoline	>128	64	>128	128	128	0.59	7.37
pergolide	>128	>128	>128	>128	>128	0.93	18.81
cabergoline	>128	>128	>128	>128	>128	0.75	17.24
methysergide	>128	>128	>128	>128	>128	1.01	15.57
S1	>128	16	>128	>128	>128	0.39	6.01
S2	>128	128	>128	>128	>128	0.85	6.94
S3	>128	>128	>128	128	>128	0.23	14.02
S4	>128	>128	>128	>128	>128	0.84	16.54
S5	>128	128	>128	64	128	0.49	14.95
S6	>128	64	128	64	64	0.08	19.86
S7	>128	32	64	64	16	0.58	17.78
S8	>128	128	>128	128	>128	0.65	17.60
S9	>128	128	>128	>128	>128	0.86	19.18
S10	>128	>128	>128	128	>128	0.87	20.78
S11	>128	32	64	>128	>128	0.56	12.64
MLBC-01	>128	32	128	8	16	0.07	18.07
MLBC-02	>128	8	32	4	0.5	0.08	8.9

[a]WT *S.* Tm was grown in MHB with 10 mM EDTA
[b]Intracellular activity was measured by addition of 8 μg mL^{-1} compound to RAW264.7 macrophages infected with WT *S.* Tm and is reported as fold-growth inhibition relative to a DMSO-treated control
[c]Toxicity was estimated by lactate dehydrogenase release from uninfected RAW264.7 cells
All numbers reflect the mean from two (MIC) or three (RAW264.7) experiments.

Supplementary Table 3. NMR data for N-acetyl ergoline derivative **S4**[a]

Position	δC, mult		δH, (mult, *J* in Hz, integration)	COSY	HMBC
1'	32.9	CH_3	3.76 (s, 3H)		C2, C15
2	122.7	CH	6.73 (s, 1H)	$H4_{eq}$, $H4_{ax}$	C1', C3, C15, C16
3	110.7	C			
4	27.1	CH_2	2.70 (dd, *J* = 14.7, 10.6 Hz, 1H, ax)	H2, $H4_{eq}$, H5	C2, C3, C5, C10
			3.38 (dd, *J* = 14.6, 4.3 Hz, 1H, eq)	H2, $H4_{ax}$, H5	C2, C3, C5, C10, C16
5	67.5	CH	2.16 (td, *J* = 10.6, 4.3 Hz, 1H)	$H4_{ax}$, $H4_{eq}$, H10	C4, C6', C7, C9, C10
6'	43.4	CH_3	2.48 (s, 3H)		C4, C5, C7, C8
7	61.6	CH_2	1.99 (dd, *J* = 11.3, 11.1 Hz, 1H, ax)	$H7_{eq}$, H8	C5, C6', C9
			3.05 (d, *J* = 11.1 Hz, 1H, eq)	$H7_{ax}$, H8	C5, C6', C8, C9
8	36.8	CH	2.07–2.13 (m, 1H)	H8'A, H8'B, $H7_{ax}$, $H7_{eq}$, $H9_{ax}$, $H9_{eq}$	C7
8'	43.5	CH_2	3.27 (m, *J* = 13.5, 6.0 Hz, 1H, A)	H8, H8'B	C7, C8, C9, C18
			3.29 (m, *J* = 13.5, 6.8 Hz, 1H, B)		
9	32.2	CH_2	1.17 (q, *J* = 12.4 Hz, 1H, ax)	H8, $H9_{eq}$, H10	C5, C7, C8, C8', C10, C11
			2.65 (m, 1H, eq)	H8, $H9_{ax}$, H10	C5, C7, C8, C10
10	40.4	CH	2.97 (ddd, *J* = 12.4, 10.6, 3.4 Hz)	H5, $H9_{ax}$, $H9_{ax}$	C5, C9, C11
11	133.3	C			
12	112.7	CH	6.89 (d, *J* = 7.1 Hz, 1H)	H13	C3, C10, C13, C14, C15, C16
13	122.8	CH	7.18 (dd, *J* = 7.1, 8.1 Hz, 1H)	H12, H14	C11, C12, C15, C16
14	107.0	CH	7.11 (d, *J* = 8.1 Hz, 1H)	H13	C11, C12, C16
15	134.5	C			
16	126.6	C			
17			5.57 (brs, 1H)	H8'A, H8'B	C18
18	170.3	C			
19	23.6	CH_3	2.03 (s, 3H)		C18

a ^{1}H NMR (700 MHz) and ^{13}C NMR (175 MHz) experiments were recorded in $CDCl_3$ at 23 °C. Positional assignments are based on HSQC and HMBC correlation experiments. Stereochemical assignments were deduced from coupling constants.

Supplementary Methods

Synthetic Experimental Procedures: General

Chemical shifts in ^{1}H NMR and ^{13}C NMR spectra are reported in parts per million (ppm) relative to tetramethylsilane (TMS), with calibration of the residual solvent peaks according to values reported by Gottlieb et al. (chloroform: δ_H 7.26, δ_C 77.16; acetone: δ_H 2.05, δ_C 29.84, 206.26; methanol: δ_H 3.31, δ_C 49.00; DMSO: δ_H 2.50, δ_C 39.52; acetonitrile: δ_H 1.94, δ_C 1.32, 118.26)[1]. When peak multiplicities are given, the following abbreviations are used: s, singlet; d, doublet; t, triplet; q, quartet; sept., septet; dd, doublet of doublets; m, multiplet; br, broad; app., apparent; *gem*, geminal. ^{1}H NMR spectra were acquired at 600 or 700 MHz with a default digital resolution (Brüker parameter: FIDRES) of 0.18 and 0.15 Hz/point, respectively. Coupling constants reported herein therefore have uncertainties of ±0.4 Hz and ±0.3 Hz, respectively. All assignments of protons and carbons relied on data from 2-dimensional NMR experiments including COSY, HMQC, and HMBC. The ^{13}C NMR (DEPTq) spectra provided herein show CH and CH_3 carbon signals below the baseline and C and CH_2 carbons above the baseline. Melting points (mp) are uncorrected. Reactions were carried out at room temperature (rt) if temperature is not specified. An automated flash chromatography system (Teledyne CombiFlash Rf 200) was used for the purification of compounds on silica gel (either 40–60 μM or 20–40 μM particle size).

General Procedure A

To a solution of amine **S1** (1.0 equiv), EDC·HCl (1.2 equiv), HOBt (1.2 equiv), and a carboxylic acid (1.2 equiv) in CH_2Cl_2 (0.02 M) was added *i*-Pr_2NEt (3 equiv). The reaction mixture was stirred at rt for 3–16 h and the progress of the reaction followed by thin-layer chromatography (TLC). Upon completion, the reaction mixture was diluted with CH_2Cl_2, washed with saturated aqueous Na_2CO_3, dried over Na_2SO_4, and concentrated under reduced pressure. Products were purified by flash chromatography (MeOH/CH_2Cl_2) on silica gel.

Aminomethyl ergoline (S1)

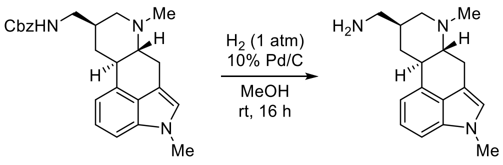

To a stirring solution of metergoline (1.00 g, 2.48 mmol) in MeOH (75 mL) was added 10% Pd/C (50 mg). The solution was degassed by stirring under vacuum for approximately 30–60 seconds, followed by back-filling with argon via balloon. After repeating the process 10 times, the flask was back-filled with H_2 gas from a balloon and the reaction stirred overnight at rt. After 14 h, the reaction vessel was purged of H_2 with argon and TLC analysis indicated the starting material was consumed. The reaction mixture was then filtered through a pad of Celite and the resulting yellow solution concentrated under reduced pressure to provide the primary amine as a viscous amber oil that was used in following reactions without further purification. The crude oil was diluted with CH_2Cl_2, aliquotted into reaction vials, and concentrated under reduced pressure. A small amount of the crude amine **S1** solidified after concentrating from CH_2Cl_2 into an amorphous solid. Mp: 135–138 °C (lit. 151–153 °C)[2]. R_f = 0.03 (10% MeOH/CH_2Cl_2). ^{1}H NMR (700 MHz, $CDCl_3$): 7.19 (dd, J = 7.1, 8.1 Hz, 1H), 7.11 (d, J = 8.1 Hz, 1H), 6.93 (d, J = 7.1 Hz, 1H), 6.72 (s, 1H), 3.76 (s, 3H), 3.39 (dd, J = 14.6, 4.2 Hz, 1H), 3.12 (d, J = 9.6 Hz, 1H), 2.97 (m, 1H), 2.75–2.65 (m, 4H), 2.48 (s, 3H), 2.14 (td, J = 10.6, 4.2 Hz, 1H), 2.00–1.90 (m, 2H), 1.10 (q, J = 12.1 Hz, 1H). ^{13}C NMR ($CDCl_3$): 27.2, 32.3, 32.9, 39.5, 40.7, 43.6, 46.8, 62.0, 67.7, 106.9, 110.9, 112.7, 122.7, 122.6, 126.7, 133.8, 134.5. LCMS (ESI) *m/z*: 270.1965 calculated for $C_{17}H_{24}N_3^+$ ($[M + H]^+$); 270.1962 observed.

Ethyl carbamate ergoline derivative (S2)

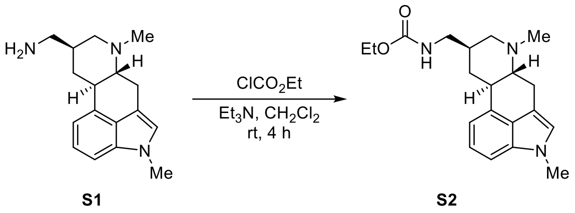

Et$_3$N (31 μL, 0.22 mmol) was added to a solution of ethyl chloroformate (31 mg, 0.14 mmol) and amine **S1** (30 mg, 0.11 mmol) in CH_2Cl_2 (5 mL) and the reaction stirred for 6 h at rt. TLC analysis at 4 h indicated that the reaction was complete, and the solution was diluted with CH_2Cl_2 (25 mL), washed with saturated Na_2CO_3 (20 mL), dried over Na_2SO_4, and concentrated under reduced pressure. Flash chromatography (0→20% MeOH/CH_2Cl_2) provided the ethyl carbamate **S2** as a yellow glassy solid (21 mg, 0.062 mmol, 56%). R_f = 0.28 (5% MeOH/CH_2Cl_2). ^{1}H NMR (700 MHz, $CDCl_3$): δ 7.18 (dd, *J* = 7.0, 8.1 Hz, 1H), 7.11 (d, *J* = 8.1 Hz, 1H), 6.90 (d, *J* = 7.0 Hz, 1H), 6.73 (s, 1H), 4.75 (brs, 1H), 4.17–4.10 (m, 2H), 3.75 (s, 3H), 3.38 (dd, *J* = 14.5, 4.2 Hz, 1H), 3.23–3.15 (m, 2H), 3.07 (d, *J* = 10.6 Hz, 1H), 3.00–2.95 (m, 1H), 2.75–2.65 (m, 2H), 2.49 (s, 3H), 2.20–2.10 (m, 2H), 2.00 (app t, *J* = 10.6 Hz, 1H), 1.26 (t, *J* = 6.8 Hz, 3H), 1.16 (app q, *J* = 12.2 Hz, 1H). ^{13}C NMR (125 MHz, $CDCl_3$): δ 156.8, 134.4, 133.2, 126.4, 122.7, 122.5, 112.6, 110.6, 106.8, 67.4, 61.3, 60.9, 44.9, 43.3, 40.4, 36.7, 32.8, 31.9, 26.9, 14.7. LCMS (ESI) *m/z*: 342.2176 calculated for $C_{20}H_{28}N_3O_2^+$ ([M + H]$^+$); 342.2203 observed.

N-Boc ergoline derivative (S3)

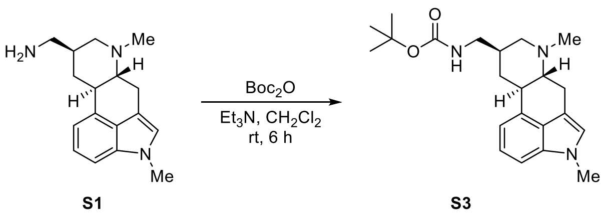

Et$_3$N (31 μL, 0.22 mmol) was added to a solution of Boc$_2$O (31 mg, 0.14 mmol) and amine **S1** (30 mg, 0.11 mmol) in CH_2Cl_2 (5 mL) and the reaction stirred for 6 h at rt. TLC analysis at 6 h indicated that the reaction was complete, and the solution was diluted with CH_2Cl_2 (25 mL), washed with saturated Na_2CO_3 (20 mL), dried over Na_2SO_4, and concentrated under reduced

pressure. Flash chromatography (0→20% MeOH/CH_2Cl_2) provided the *t*-butyl carbamate **S3** as a viscous oily solid (32 mg, 0.087 mmol, 86%). R_f = 0.26 (5% MeOH/CH_2Cl_2). ^{1}H NMR (700 MHz, $CDCl_3$): δ 7.18 (dd, *J* = 7.1, 8.1 Hz, 1H), 7.11 (d, *J* = 8.1 Hz, 1H), 6.90 (d, *J* = 7.1 Hz, 1H), 6.73 (s, 1H), 4.70–4.59 (brs, 1H), 3.75 (s, 3H), 3.38 (dd, *J* = 14.5, 4.2 Hz, 1H), 3.23–3.16 (m, 1H), 3.12–3.02 (m, 2H), 2.95 (m, 1H), 2.72–2.64 (m, 2H), 2.47 (s, 3H), 2.16–2.11 (m, 1H) 2.11–2.06 (m, 1H), 1.96 (app t, *J* = 10.9 Hz, 1H), 1.46 (s, 9H), 1.14 (app q, *J* = 12.2 Hz, 1H). ^{13}C NMR (175 MHz, $CDCl_3$): δ 156.1, 134.4, 133.3, 126.5, 122.7, 122.5, 112.5, 110.7, 106.8, 79.3, 67.4, 61.5, 44.6, 43.4, 40.5, 36.9, 32.8, 32.0, 28.4 (3C), 27.0. LCMS (ESI) *m/z*: 370.2489 calculated for $C_{22}H_{32}N_3O_2^+$ ($[M + H]^+$); 370.2526 observed.

***N*-Acetyl ergoline derivative (S4)**

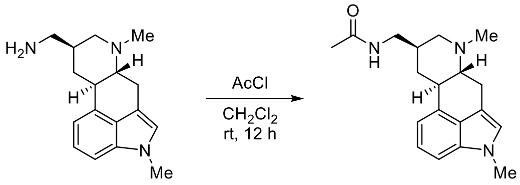

S1 S4

In a modification of a known procedure,[2] acetyl chloride (16 μL, 0.22 mmol) was added to a solution of amine **S1** (30 mg, 0.11 mmol) in CH_2Cl_2 (5 mL) and stirred overnight at rt. After 12 h, the reaction mixture was diluted with CH_2Cl_2 (25 mL), washed with saturated Na_2CO_3 (20 mL), dried over Na_2SO_4, and concentrated under reduced pressure to give a yellow oil that solidified under vacuum. Flash chromatography (0→20% MeOH/CH_2Cl_2) provided acetamide **S4** (30 mg, 0.10 mmol, 88%) as an amorphous yellow solid. R_f = 0.08 (5% MeOH/CH_2Cl_2). Mp: 191–194 °C (lit. 191–193 °C)[2]. ^{1}H NMR (700 MHz, $CDCl_3$): 7.18 (dd, *J* = 7.1, 8.2 Hz, 1H), 7.11 (d, *J* = 8.2 Hz, 1H), 6.89 (d, *J* = 7.1 Hz, 1H), 6.73 (s, 1H), 5.59 (brs, 1H), 3.76 (s, 3H), 3.38 (dd, *J* = 14.6, 4.3 Hz, 1H), 3.29 (A of ABX, *J* = 13.5, 6.8 Hz, 1H), 3.27 (B of ABX, *J* = 13.5, 6.0 Hz, 1H), 3.05 (d, *J* = 11.2 Hz, 1H), 2.97 (ddd, *J* = 12.3, 10.6, 3.4 Hz, 1H), 2.65–2.72 (m, 2H), 2.48 (s, 3H), 2.16 (td, *J* = 10.6 Hz, *J* = 4.3 Hz, 1H), 2.07–2.15 (m, 1H), 2.03 (s, 3H), 1.99 (dd, *J* = 11.3, 11.1 Hz, 1H), 1.17 (q, *J* = 12.3 Hz, 1H). ^{13}C NMR (150 MHz, $CDCl_3$): 170.2, 134.4, 133.1, 126.4, 122.7, 122.6, 112.5, 110.5, 106.9, 67.4. 61.4, 43.4, 43.3, 40.5, 36.6, 32.8, 32.1, 26.9, 23.4. LCMS (ESI) *m/z*: 312.2070 calculated for $C_{19}H_{26}N_3O^+$ ($[M + H]^+$); 312.2076 observed.

N-(*N*'-Benzyl) urea derivative (S5)

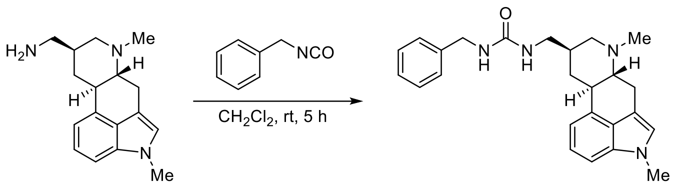

S1 **S5**

Benzyl isocyanate (15 μL, 0.12 mmol) was added to a solution of amine **S1** (30 mg, 0.11 mmol) in CH_2Cl_2 (5 mL) and stirred at rt for 5 h. The reaction mixture was diluted with CH_2Cl_2 (25 mL), washed with saturated Na_2CO_3 (20 mL), dried over Na_2SO_4, and concentrated under reduced pressure. Flash chromatography (0→10% MeOH/CH_2Cl_2) provided the *N*-benzyl urea derivative **S5** as a beige solid (28 mg, 0.069 mmol, 63%). R_f = 0.12 (5% MeOH/CH_2Cl_2). Mp: sintered at 200–205 °C; melted at 265–270 °C (dec). ^{1}H NMR (700 MHz, $CDCl_3$): δ 7.32–7.34 (t, 3H), 7.23–7.26 (m, 2H), 7.18 (dd, *J* = 7.1, 8.1 Hz, 2H), 7.11 (d, *J* = 8.1 Hz, 1H), 6.86 (d, *J* = 7.1 Hz, 1H), 6.72 (s, 1H), 4.87 (brs, 1H), 4.61 (brs, 1H), 4.41 (A or ABX, *J* = 15.0, 5.9 Hz, 1H), 4.37 (B or ABX, *J* = 15.0, 5.8 Hz, 1H), 3.75 (s, 3H), 3.37 (dd, *J* = 14.5, 4.2 Hz, 1H), 3.20 (m, 1H), 3.06 (brd, *J* = 10.6 Hz, 1H), 3.02–2.98 (m, 1H), 2.73 (app t, *J* = 12.4, Hz, 1H), 2.62 (d, *J* = 12.0 Hz, 1H), 2.48 (s, 3H), 2.20 (td, *J* = 10.6 Hz, *J* = 4.0 Hz, 1H), 2.15–2.05 (m, 1H), 2.00 (t, *J* = 11.4 Hz, 1H), 1.12 (q, *J* = 12.3 Hz, 1H). ^{13}C NMR (175 MHz, $CDCl_3$): δ 158.2, 139.2, 134.4, 132.9, 128.7 (2C), 127.5 (2C), 127.4, 126.4, 122.7, 122.6, 112.6, 110.2, 106.9, 67.4, 31.3, 44.7, 44.3, 43.1, 40.2, 36.8, 32.8, 31.9, 26.7. LCMS (ESI) *m/z*: 403.2492 calculated for $C_{25}H_{31}N_4O^+$ ([M + H]$^+$); 403.2565 observed.

N-(2-Phenylethylsulfonyl) ergoline derivative (S6)

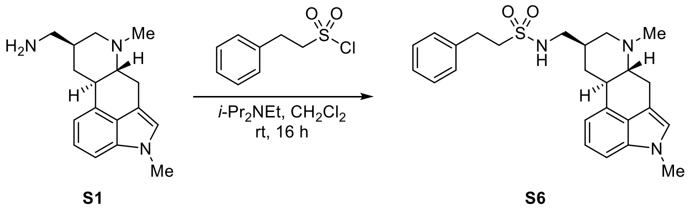

S1 **S6**

To a solution of amine **S1** (30 mg, 0.11 mmol) and 2-phenylethanesulfonyl chloride (26 mg, 0.13 mmol) in CH_2Cl_2 (5 mL) was added *i*-Pr_2NEt (58 μL, 0.33 mmol). After stirring the reaction mixture at rt for 5 h, it was diluted with CH_2Cl_2 (25 mL), washed with saturated Na_2CO_3 (20 mL),

dried over Na_2SO_4, and concentrated under reduced pressure to give a yellow oily solid. Flash chromatography (0→10% MeOH/CH_2Cl_2) provided sulfonamide **S6** as a pale yellow amorphous solid (27 mg, 0.062 mmol, 56%). R_f = 0.24 (5% MeOH/CH_2Cl_2). ^{1}H NMR (600 MHz, $CDCl_3$): 7.35 (t, *J* = 7.5 Hz, 2H), 7.29–7.25 (m, 3H), 7.18 (dd, *J* = 8.2, 7.1 Hz, 1H), 7.12 (d, *J* = 8.2 Hz, 1H), 6.86 (d, *J* = 7.1 Hz, 1H), 6.73 (s, 1H), 4.10 (brs, 1H), 3.75 (s, 3H), 3.37 (dd, *J* = 14.7, 4.4 Hz, 1H), 3.35–3.31 (m, 2H), 3.18–3.14 (m, 1H), 3.05 (d, *J* = 10.6 Hz, 1H), 3.02–2.90 (m, 3H), 2.72–2.66 (m, 1H), 2.64–2.58 (m, 1H), 2.47 (s, 3H), 2.13 (td, *J* = 10.8 Hz, *J* = 4.3 Hz, 1H), 1.92 (t, *J* = 11.3 Hz, 1H), 1.08 (q, *J* = 12.3 Hz, 1H). ^{13}C NMR (150 MHz, $CDCl_3$): 138.0, 134.5, 133.0, 129.2 (2C), 128.6 (2C), 127.3, 126.6, 122.9, 122.7, 112.6, 110.6, 107.0, 67.5, 61.2, 53.7, 47.4, 43.4, 40.5, 36.5, 32.9, 32.0, 30.3, 27.0. LCMS (ESI) *m/z*: 438.2210 calculated for $C_{25}H_{32}N_3O_2S^+$ ([M + H]$^+$); 438.2301 observed.

***N*-(3-Phenylpropyl) ergoline derivative (S7)**

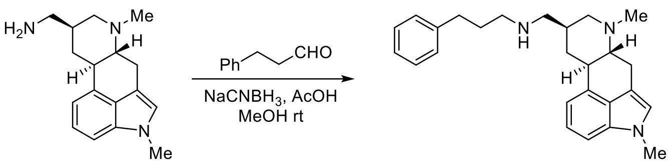

Amine **S1** (36 mg, 0.13 mmol) and 3-phenylpropanal (20 mg, 0.15 mmol) were combined and stirred with Na_2SO_4 (50 mg) in MeOH (5 mL) at rt. After stirring for 15 min, $NaCNBH_3$ (51 mg, 0.82 mmol) and AcOH (50 μL) were added and the solution stirred at rt for 2 h. The reaction mixture was diluted with CH_2Cl_2 (50 mL), washed with saturated Na_2CO_3 (30 mL), dried over Na_2SO_4, and concentrated under reduced pressure. Flash chromatography (0→20% MeCN/H_2O) provided amine **S7** as an off-white amorphous solid (32 mg, 0.083 mmol, 63%). R_f = 0.10 (10% MeOH/CH_2Cl_2). ^{1}H NMR (600 MHz, $CDCl_3$): 7.28–7.24 (m, 2H), 7.20–7.15 (m, 4H), 7.09 (d, *J* = 8.2 Hz, 1H), 6.92 (d, *J* = 7.1 Hz, 1H), 6.70 (s, 1H), 3.73 (s, 3H), 3.36 (dd, *J* = 14.6, 4.3 Hz, 1H), 3.22 (brd, *J* = 10.4 Hz, 1H), 3.01 (brt, *J* = 10.4, 3.5 Hz, 1H), 2.78–2.73 (m, 4H), 2.72–2.61 (m, 5H), 2.50 (s, 3H), 2.30–2.22 (m, 1H), 2.21 (td, *J* = 10.7, 4.2 Hz, 1H), 2.02 (t, *J* = 11.7 Hz, 1H), 1.99–1.97 (m, 2H), 1.11 (q, *J* = 12.3 Hz, 1H). ^{13}C NMR (150 MHz, $CDCl_3$): 141.5, 134.3, 133.0, 128.4 (4C), 126.4, 125.9, 122.7, 122.5, 112.7, 110.3, 106.8, 67.4, 61.7, 53.3, 49.0, 43.0, 40.2, 34.9, 33.5, 32.8, 32.5, 30.4, 26.6. LCMS (ESI) *m/z*: 388.2747 calculated for

$C_{24}H_{34}N_3^+$ ([M + H]$^+$); 388.2785 observed.

N-Benzoyl ergoline derivative (S8)

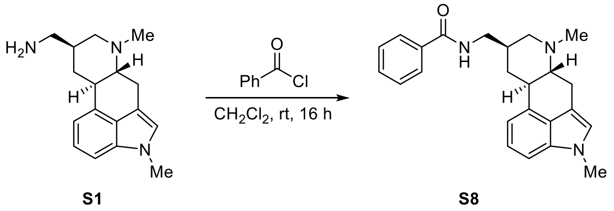

Benzoyl chloride (20 mg, 0.15 mmol) was added to a solution of the amine **S1** (30 mg, 0.11 mmol) in CH_2Cl_2 (5 mL) and stirred overnight at rt. After 16 h, the reaction mixture was diluted with CH_2Cl_2 (25 mL), washed with saturated Na_2CO_3 (20 mL), dried over Na_2SO_4, and concentrated under reduced pressure. Flash chromatography (0→20% MeOH/CH_2Cl_2) provided amide **S8** as an amber oily solid (32 mg, 0.085 mmol, 77%). R_f = 0.20 (5% MeOH/CH_2Cl_2). ^{1}H NMR (700 MHz, $CDCl_3$): 7.82 (d, *J* = 7.3 Hz, 2H), 7.51 (t, *J* = 7.4 Hz, 1H), 7.45 (dd, *J* = 7.3, 7.4 Hz, 2H), 7.19 (dd, *J* = 7.1, 8.2 Hz, 1H), 7.13 (d, *J* = 8.2 Hz, 1H), 6.90 (d, *J* = 7.1 Hz, 1H), 6.74 (s, 1H), 6.50–6.44 (brs, 1H), 3.75 (s, 3H), 3.55–3.45 (m, 2H), 3.40 (dd, *J* = 14.6, 4.3 Hz, 1H), 3.21 (d, *J* = 12.4 Hz, 1H), 3.14–3.07 (m, 1H), 2.85–2.75 (m, 1H), 2.74 (d, *J* = 12.6 Hz, 1H), 2.54 (s, 3H), 2.39–2.33 (m, 1H), 2.30 (td, *J* = 4.4, 10.8 Hz, 1H), 2.16 (t, *J* = 11.4 Hz, 1H), 1.31 (q, *J* = 12.4 Hz, 1H). ^{13}C NMR (150 MHz, $CDCl_3$): 167.8, 134.6, 134.4, 132.6, 131.5, 128.6 (2C), 126.9 (2C), 126.4, 122.8, 122.7, 112.6, 110.1, 107.0, 67.4. 61.3, 43.6, 43.0, 40.2, 36.4, 32.8, 32.0, 26.6. LCMS (ESI) *m/z*: 374.2227 calculated for $C_{24}H_{28}N_3O^+$ ([M + H]$^+$); 374.2296 observed.

N-Phenylacetyl ergoline derivative (S9)

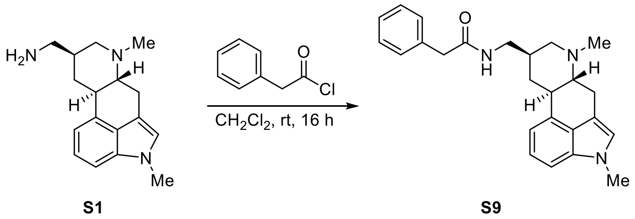

Phenylacetyl chloride (22 µL, 0.17 mmol) was added to a solution of the amine **S1** (30 mg, 0.11 mmol) in CH_2Cl_2 (5 mL) and stirred overnight at rt. After 16 h, the reaction mixture was diluted with CH_2Cl_2 (25 mL), washed with saturated Na_2CO_3 (20 mL), dried over Na_2SO_4, and

concentrated under reduced pressure. Flash chromatography (0→20% MeOH/CH_2Cl_2) provided amide **S9** as an amorphous pale yellow solid (20 mg, 0.050 mmol, 45%). R_f = 0.22 (5% MeOH/CH_2Cl_2). ^{1}H NMR (700 MHz, $CDCl_3$): 7.38–7.36 (t, J = 7.5 Hz, 2H), 7.34–7.28 (m, 3H), 7.18 (dd, J = 7.1, 8.2 Hz, 1H), 7.12 (d, J = 8.2 Hz, 1H), 6.81 (d, J = 7.1 Hz, 1H), 6.73 (s, 1H), 5.75–5.68 (brs, 1H), 3.75 (s, 3H), 3.60 (s, 2H), 3.37 (dd, J = 14.5, 4.3 Hz, 1H), 3.31–3.26 (m, 1H), 3.22–3.17 (m, 1H), 3.14–3.04 (m, 2H), 2.86 (app t, J = 12.7 Hz, 1H), 2.57–2.53 (m, 1H), 2.55 (s, 3H), 2.38–2.33 (m, 1H), 2.24–2.16 (m, 1H), 2.09 (t, J = 11.4 Hz, 1H), 1.13 (q, J = 12.4 Hz, 1H). ^{13}C NMR (175 MHz, $CDCl_3$): 171.3, 135.0, 134.3, 132.0, 129.5 (2C), 129.1 (2C), 128.3, 127.4, 126.2, 122.8, 112.6, 109.5, 107.1, 67.3. 60.7, 43.9, 42.9, 42.5, 39.6, 35.7, 32.8, 31.5, 26.1. LCMS (ESI) *m/z*: 388.2383 calculated for $C_{25}H_{30}N_3O^+$ ($[M + H]^+$); 388.2448 observed.

N-(3-Phenylpropanoyl) ergoline derivative (S10)

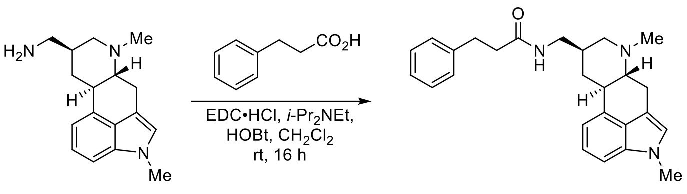

According to General Procedure A, *i*-Pr_2NEt (58 μL, 0.33 mmol) was added to a solution of amine **S1** (30 mg, 0.11 mmol), EDC·HCl (25 mg, 0.13 mmol), HOBt (18 mg, 0.13 mmol), and hydrocinnamic acid (20 mg, 0.13 mmol) in CH_2Cl_2 (5 mL). After stirring the reaction mixture overnight at rt for 16 h, it was diluted with CH_2Cl_2 (25 mL), washed with saturated Na_2CO_3 (20 mL), dried over Na_2SO_4, and concentrated under reduced pressure to give a yellow solid. Flash chromatography (0→15% MeOH/CH_2Cl_2) provided amide **S10** as a pale yellow oil that later solidified (36 mg, 0.089 mmol, 81%). R_f = 0.19 (5% MeOH/CH_2Cl_2). ^{1}H NMR (700 MHz, $CDCl_3$): 7.29 (t, J = 7.6 Hz, 2H), 7.23 (d, J = 7.1 Hz, 2H), 7.20–7.16 (m, 2H), 7.12 (d, J = 8.2 Hz, 1H), 6.85 (d, J = 7.0 Hz, 1H), 6.73 (s, 1H), 5.41 (brs, 1H), 3.75 (s, 3H), 3.37 (dd, J = 14.6, 4.3 Hz, 1H), 3.27 (dt, J = 13.7, 6.9 Hz, 1H), 3.22–3.16 (m, 1H), 3.02–2.97 (m, 2H), 2.94–2.87 (m, 2H), 2.70–2.64 (m, 1H), 2.57–2.47 (m, 3H), 2.44 (s, 3H), 2.11 (td, J = 10.8 Hz, J = 4.3 Hz, 1H), 2.05–1.95 (m, 1H), 1.89 (t, J = 11.3 Hz, 1H), 1.08 (q, J = 12.3 Hz, 1H). ^{13}C NMR (175 MHz, $CDCl_3$): 172.2, 140.9, 134.5, 133.3, 128.7 (2C), 128.5 (2C), 126.6, 126.5, 122.8, 122.7, 112.7, 110.8, 107.0, 67.5. 61.5, 43.5, 43.4, 40.6, 38.7, 36.7, 32.9, 32.2, 31.9, 27.1. LCMS (ESI) *m/z*: 402.2540

calculated for $C_{26}H_{32}N_3O^+$ ([M + H]$^+$); 402.2536 observed.

***N*-(*N*-Boc-L-Phenylalanyl) ergoline derivative (S12)**

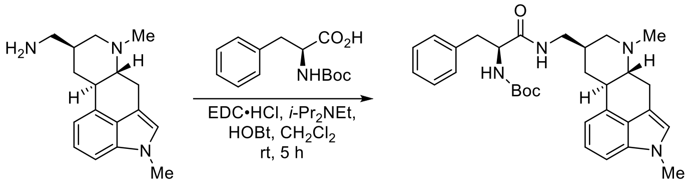

S1 **S12**

According to General Procedure A, *i*-Pr_2NEt (117 µL, 0.67 mmol) was added to a solution of amine **S1** (60 mg, 0.22 mmol), EDC·HCl (51 mg, 0.27 mmol), HOBt (36 mg, 0.27 mmol), and *N*-Boc-L-phenylalanine (65 mg, 0.13 mmol) in CH_2Cl_2 (10 mL). After stirring the reaction mixture at rt for 3.5 h, it was diluted with CH_2Cl_2 (25 mL), washed with saturated Na_2CO_3 (20 mL), dried over Na_2SO_4, and concentrated under reduced pressure to give a yellow oily solid. Flash chromatography (0→10% MeOH/CH_2Cl_2) provided the *N*-Boc-Phe derivative **S12** as an off-white solid (69.3 mg, 0.13 mmol, 60%). R_f = 0.19 (5% MeOH/CH_2Cl_2). Mp: 210–213 °C. ^{1}H NMR (700 MHz, $CDCl_3$): δ 7.31 (t, *J* = 7.5 Hz, 2H), 7.24 (d, *J* = 7.3 Hz, 2H), 7.22–7.16 (m, 2H), 7.11 (d, *J* = 8.1 Hz, 1H), 6.82 (d, *J* = 7.0 Hz, 1H), 6.73 (s, 1H), 5.79 (brs, 1H), 5.10 (brs, 1H), 4.31 (brs, 1H), 3.76 (s, 3H), 3.36 (dd, *J* = 14.6, 4.2 Hz, 1H), 3.23 (dt, *J* = 12.9, 6.2 Hz, 1H), 3.15–3.08 (m, 2H), 3.08–3.00 (m, 1H), 2.89–2.80 (m, 2H), 2.65 (app t, *J* = 12.4, Hz, 1H), 2.42 (s, 3H), 2.08 (td, *J* = 10.5 Hz, *J* = 4.0 Hz, 1H), 2.00–1.91 (m, 1H), 1.83 (t, *J* = 11.1 Hz, 1H), 1.00 (q, *J* = 12.2 Hz, 1H). ^{13}C NMR (175 MHz, $CDCl_3$): δ 171.3, 155.6, 137.0, 134.5, 133.3, 129.4 (4C), 128.9, 127.3, 126.6, 122.8, 122.6, 112.8, 110.8, 106.9, 80.4, 67.4, 61.4, 43.5, 43.4, 40.6, 38.9, 36.3, 32.9, 32.1, 28.5 (3C), 27.1. LCMS (ESI) *m/z*: 517.3173 calculated for $C_{31}H_{41}N_4O_3^+$ ([M + H]$^+$); 517.3384 observed.

N-L-Phenylalanine ergoline derivative (S11)

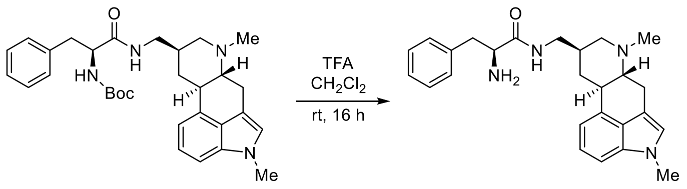

S1 **S11**

TFA (3 mL) was added to a stirring solution of the Boc-protected Phe derivative **S1** (49 mg, 0.12 mmol) in CH_2Cl_2 (10 mL) and the reaction mixture stirred at rt for 2.5 h. The reaction mixture was concentrated under reduced pressure, redissolved in CH_2Cl_2 (25 mL), and washed with saturated Na_2CO_3 (25 mL). The organic solution was dried over Na_2SO_4, concentrated under reduced pressure, and flash chromatography (0→25% MeOH/CH_2Cl_2) provided the free amine **S11** as a white amorphous solid (40 mg, 0.095 mmol, 80%). R_f = 0.30 (10% MeOH/CH_2Cl_2). ^{1}H NMR (600 MHz, $CDCl_3$): 7.47–7.43 (m, 1H), 7.33 (t, *J* = 7.6 Hz, 2H), 7.27–7.24 (m, 3H), 7.19 (dd, *J* = 7.1, 8.2 Hz, 1H), 7.12 (d, *J* = 8.2 Hz, 1H), 6.85 (d, *J* = 7.1 Hz, 1H), 6.73 (s, 1H), 3.75 (s, 3H), 3.64 (dd, *J* = 9.2, 4.1 Hz, 1H), 3.38 (dd, *J* = 14.6, 4.3 Hz, 1H), 3.33–3.27 (m, 3H), 3.03 (brd, *J* = 11.2 Hz, 1H), 3.00–2.96 (m, 1H), 2.75–2.67 (m, 2H), 2.63 (brd, *J* = 12.8 Hz, 1H), 2.49 (s, 3H), 2.17 (td, *J* = 10.7 Hz, *J* = 4.3 Hz, 1H), 2.15–2.10 (m, 1H), 2.00 (t, *J* = 11.4 Hz, 1H), 1.08 (q, *J* = 12.4 Hz, 1H). ^{13}C NMR (150 MHz, $CDCl_3$): 174.4, 137.9, 134.4, 133.1, 129.3 (2C), 128.8 (2C), 126.9, 126.4, 122.7, 122.6, 112.6, 110.5, 106.9, 67.4. 61.4, 56.6, 43.3, 42.8, 41.1 40.4, 36.5, 32.8, 32.0, 26.8. LCMS (ESI) *m/z*: 417.2649 calculated for $C_{26}H_{33}N_4O^+$ ([M + H]$^+$); 417.2723 observed.

N-trans-Cinnamoyl ergoline derivative (MLBC-01)

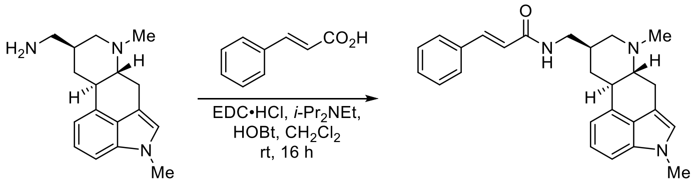

S1 **MLBC-01**

According to General Procedure A, *i*-Pr_2NEt (58 μL, 0.33 mmol) was added to a solution of amine **S1** (30 mg, 0.11 mmol), EDC·HCl (25 mg, 0.13 mmol), HOBt (18 mg, 0.13 mmol), and *trans*-cinnamic acid (20 mg, 0.14 mmol) in CH_2Cl_2 (5 mL). After stirring the reaction mixture at rt

for 5 h, it was diluted with CH_2Cl_2 (25 mL), washed with saturated Na_2CO_3 (20 mL), dried over Na_2SO_4, and concentrated under reduced pressure to give a yellow oily solid. Flash chromatography (0→15% MeOH/CH_2Cl_2) provided the *N*-cinnamoyl derivative **MLBC-01** as a pale yellow amorphous solid (31 mg, 0.078 mmol, 71%). R_f = 0.24 (5% MeOH/CH_2Cl_2). ^{1}H NMR (700 MHz, $CDCl_3$): 7.66 (d, *J* = 15.6 Hz, 1H), 7.52 (d, *J* = 6.7 Hz, 1H), 7.40–7.34 (m, 3H), 7.18 (dd, *J* = 7.1, 8.2 Hz, 1H), 7.11 (d, *J* = 8.2 Hz, 1H), 6.91 (d, *J* = 7.1 Hz, 1H), 6.73 (s, 1H), 6.43 (d, *J* = 15.6 Hz, 1H), 5.73 (brs, 1H), 3.76 (s, 3H), 3.48–3.40 (m, 2H), 3.39 (dd, *J* = 14.6, 4.3 Hz, 1H), 3.10 (app d, *J* = 11.1 Hz, 1H), 3.02–1.98 (m, 1H), 2.75–2.67 (m, 2H), 2.49 (s, 3H), 2.23–2.19 (brs, 1H), 2.19 (td, *J* = 10.6 Hz, *J* = 4.2 Hz, 1H), 2.06 (app t, *J* = 11.7 Hz, 1H), 1.23 (q, *J* = 12.4 Hz, 1H). ^{13}C NMR (175 MHz, $CDCl_3$): 166.2, 141.4, 135.0, 134.5, 133.3, 129.8, 129.0 (2C), 128.0 (2C), 126.6, 122.9, 122.7, 120.7, 112.7, 10.8, 107.0, 67.5, 61.6, 43.6, 43.5, 40.7, 37.0, 32.9, 32.3, 27.1. LCMS (ESI) *m/z*: 400.2383 calculated for $C_{26}H_{32}N_3O^+$ ($[M + H]^+$); 400.2438 observed.

N-(4-Chloro-*trans*-cinnamoyl) ergoline derivative (MLBC-02)

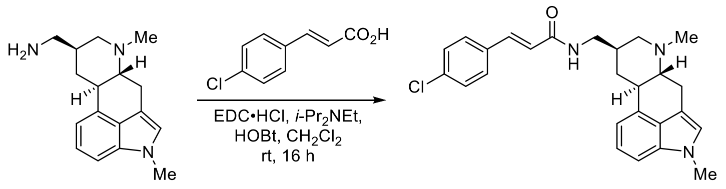

S1 **MLBC-02**

According to General Procedure A, *i*-Pr_2NEt (194 μL, 1.11 mmol) was added to a solution of amine **S1** (100 mg, 0.37 mmol), EDC·HCl (85 mg, 0.44 mmol), HOBt (60 mg, 0.44 mmol), and 4-chlorocinnamic acid (81 mg, 0.44 mmol) in CH_2Cl_2 (10 mL). After stirring the reaction mixture overnight at rt for 16 h, it was diluted with CH_2Cl_2 (50 mL), washed with saturated Na_2CO_3 (2 × 40 mL), dried over Na_2SO_4, and concentrated under reduced pressure to give an off-white solid. Flash chromatography (0→15% MeOH/CH_2Cl_2) provided amide **MLBC-02** as a white solid (133 mg, 0.31 mmol, 83%). ^{1}H NMR (700 MHz, $CDCl_3$): 7.60 (d, *J* = 15.6 Hz, 1H, H20), 7.44 (d, *J* = 8.1 Hz, 2H, H22), 7.34 (d, *J* = 8.1 Hz, 2H, H23), 7.18 (dd, *J* = 8.2, 7.1 Hz, 1H, H13), 7.11 (d, *J* = 8.2 Hz, 1H, H14), 6.89 (d, *J* = 7.1 Hz, 1H, H12), 6.73 (s, 1H, H2), 6.42 (d, *J* = 15.6 Hz, 1H, H19), 5.94–5.87 (brs, 1H, NH), 3.76 (s, 3H, C1′), 3.46 (dt, *J* = 14.1, 7.2 Hz, 1H, H8′A), 3.43–3.36 (m, 2H, H8′B + $H4_{eq}$), 3.16 (app d, *J* = 11.2, 1H, $H7_{eq}$), 3.08 (brt, *J* = 10.6, 3.1 Hz, 1H, H10), 2.78 (app t, *J* = 12.8 Hz, 1H, $H4_{ax}$), 2.72 (brd, *J* = 10.6, 3.1 Hz, 1H, $H9_{eq}$), 2.54 (s, 3H), 2.32–2.23 (m,

2H, H5 + H8), 2.13 (app t, J = 11.2 Hz, 1H, $H7_{ax}$), 1.24 (app q, J = 12.4 Hz, 1H, $H9_{ax}$). ^{13}C NMR (150 MHz, $CDCl_3$): 166.0, 140.1, 135.7, 134.5, 133.4, 132.8, 129.2 (2C), 129.1 (2C), 126.5, 122.9, 122.8, 121.2, 112.7, 110.2 107.1, 67.6, 61.4, 43.5, 43.2, 40.3, 36.6, 33.0, 32.1, 26.8. LCMS (ESI) *m/z*: 434.1994 calculated for $C_{26}H_{29}{}^{35}Cl\,N_3O^+$ ([M + H]$^+$); 434.21842 observed.

Supplementary References

1. Gottlieb HE, Kotlyar V, Nudelman A. NMR Chemical Shifts of Common Laboratory Solvents as Trace Impurities. *J Org Chem* **62**, 7512-7515 (1997).

2. Singh K, Kaur G, Mjambili F, Smith PJ, Chibale K. Synthesis of metergoline analogues and their evaluation as antiplasmodial agents. *MedChemComm* **5**, 165-170 (2014).
