# Supplementary material for: A macrophage-based screen identifies antibacterial compounds selective for intracellular Salmonella Typhimurium
Source: Nat Commun. 2019 Jan 14;10:197. doi: 10.1038/s41467-018-08190-x (PMC6331611; doi:10.1038/s41467-018-08190-x)
Supplement: Supplementary file 3 — Description of Additional Supplementary Files [file 41467_2018_8190_MOESM3_ESM.pdf]

Description of Additional Supplementary Files

File Name: Supplementary Data 1
Description: Growth of the *S.* Tm Single Gene Deletion (SGD) collection in LPM. The SGD was grown in triplicate for 16 h in LPM and OD_{600} was measured for each of the 3,724 mutants. Normalized growth of each mutant is shown. This data is related to Figure 1A

File Name: Supplementary Data 2
Description: Intracellular replication of select SGD mutants in RAW264.7 macrophages. Mutants from the SGD were used to infect RAW264.7 macrophages and CFUs were enumerated after 7 h. The average fold-change in CFU for technical duplicates (between T0 and T7) is presented. Mutants are categorized as important for growth in LPM, motility genes, regulatory genes, SPI-1 or SPI-2 genes, and miscellaneous virulence genes. This data is related to Figure 1B.

File Name: Supplementary Data 3
Description: Small molecule screening data for LPM and intra-macrophage screens. The normalized growth of *S.* Tm exposed to each compound in the 1600 molecule Pharmakon library is presented: for the LPM screen this refers to OD600 measurements and for RAW264.7 macrophage screens this refers to luminescence. Values for each screening replicate are shown. This data is related to Figure 2A.

File Name: Supplementary Data 4
Description: Minimum inhibitory concentrations (MIC, µM) and relative cytotoxicity for primary screen actives. The MIC for each compound against *S.* Tm grown in MHB, MOPS glucose minimal media, or LPM is the concentration that resulted in no detectable growth after 16 h. The macrophage MIC is estimated as the concentration of compound that reduced luminescence ≥10-fold. Cytotoxicity reports lactate dehydrogenase release from uninfected macrophages exposed to 50 µM compound for 2 h as a percentage relative to controls. All values reflect the mean of duplicate measurements. This data is related to Figure 2B.

File Name: Supplementary Data 5
Description: Media selectivity for compounds with a minimum inhibitory concentration ≤ 3 µM. The activity of the most potent compounds was compared across all conditions. Compounds are grouped in columns based on the media in which the MIC is ≤ 3 µM. This data is related to Figure 2B and Supplementary Data 4.

File Name: Supplementary Data 6
Description: Growth of the *Salmonella* Single Gene Deletion (SGD) collection in MHB or MHB with 100 µg mL^{-1} metergoline. The SGD was grown for 16 h in MHB or MHB + 100 µg mL^{-1} metergoline in duplicate. The normalized growth of each mutant is shown. This data is related to Figure 3A.

File Name: Supplementary Data 7
Description: Analysis of *S.* Tm surface characteristics under different conditions. Roughness and Power Spectral Density (PSD) quantitation from Atomic Force Microscopy (AFM) is shown. AFM images of *S.* Tm grown in MHB, LPM, or isolated directly from bone-marrow derived murine macrophages were quantified using NanoScope software (Bruker). This data is related to Figure 4.
