# Supplementary material for: A macrophage-based screen identifies antibacterial compounds selective for intracellular Salmonella Typhimurium
Source: Nat Commun. 2019 Jan 14;10:197. doi: 10.1038/s41467-018-08190-x (PMC6331611; doi:10.1038/s41467-018-08190-x)
Supplement: Supplementary file 11 — Reporting Summary [file 41467_2018_8190_MOESM11_ESM.pdf]

nature research

Corresponding author(s): Brian K. Coombes and Eric D. Brown

Reporting Summary

Nature Research wishes to improve the reproducibility of the work that we publish. This form provides structure for consistency and transparency in reporting. For further information on Nature Research policies, see Authors & Referees and the Editorial Policy Checklist.

Statistical parameters

When statistical analyses are reported, confirm that the following items are present in the relevant location (e.g. figure legend, table legend, main text, or Methods section).

n/a	Confirmed	
☐	☒	The exact sample size (*n*) for each experimental group/condition, given as a discrete number and unit of measurement
☐	☒	An indication of whether measurements were taken from distinct samples or whether the same sample was measured repeatedly
☐	☒	The statistical test(s) used AND whether they are one- or two-sided<br>*Only common tests should be described solely by name; describe more complex techniques in the Methods section.*
☒	☐	A description of all covariates tested
☐	☒	A description of any assumptions or corrections, such as tests of normality and adjustment for multiple comparisons
☐	☒	A full description of the statistics including central tendency (e.g. means) or other basic estimates (e.g. regression coefficient) AND variation (e.g. standard deviation) or associated estimates of uncertainty (e.g. confidence intervals)
☐	☒	For null hypothesis testing, the test statistic (e.g. *F*, *t*, *r*) with confidence intervals, effect sizes, degrees of freedom and *P* value noted<br>*Give P values as exact values whenever suitable.*
☒	☐	For Bayesian analysis, information on the choice of priors and Markov chain Monte Carlo settings
☒	☐	For hierarchical and complex designs, identification of the appropriate level for tests and full reporting of outcomes
☒	☐	Estimates of effect sizes (e.g. Cohen's *d*, Pearson's *r*), indicating how they were calculated
☐	☒	Clearly defined error bars<br>*State explicitly what error bars represent (e.g. SD, SE, CI)*

Our web collection on statistics for biologists may be useful.

Software and code

Policy information about availability of computer code

Data collection	No software was used.
Data analysis	Data were analyzed using RStudio version 1.0.143 with R version 3.2.2, GraphPad Prism 6.0 software (GraphPad Inc., San Diego, CA).

For manuscripts utilizing custom algorithms or software that are central to the research but not yet described in published literature, software must be made available to editors/reviewers upon request. We strongly encourage code deposition in a community repository (e.g. GitHub). See the Nature Research guidelines for submitting code & software for further information.

Data

Policy information about availability of data

All manuscripts must include a data availability statement. This statement should provide the following information, where applicable:

- Accession codes, unique identifiers, or web links for publicly available datasets
- A list of figures that have associated raw data
- A description of any restrictions on data availability

The source data underlying Figs 1a-b, 2a-b, 3a, 4 are provided as Supplementary Data files 1-7

Field-specific reporting

Please select the best fit for your research. If you are not sure, read the appropriate sections before making your selection.

☒ Life sciences ☐ Behavioural & social sciences ☐ Ecological, evolutionary & environmental sciences

For a reference copy of the document with all sections, see nature.com/authors/policies/ReportingSummary-flat.pdf

Life sciences study design

All studies must disclose on these points even when the disclosure is negative.

Sample size	No assumptions regarding sample or effect size were made. Sample sizes were selected based on results from pilot experiments.
Data exclusions	No data were excluded from any analyses other than the animal experiments reported in Figure 6A. In this case, one single animal was excluded due to bacterial counts that were significantly inconsistent (in all organs) with all others, as was determined with the Grubbs' outliers test.
Replication	For all follow-up in vitro experiments (i.e. not including any genome-wide or chemical screens), results were independently evaluated with at least 3 (in one instance, 2) biological replicates. All animal experiments were performed three independent times and a minimum of 3 mice were included per group.
Randomization	For animal experiments, mice were relocated at random from a housing cage to treatment or control cages.
Blinding	Blinding was deemed unnecessary for this study.

Reporting for specific materials, systems and methods

Materials & experimental systems

n/a	Involved in the study	
☒	☐	Unique biological materials
☒	☐	Antibodies
☐	☒	Eukaryotic cell lines
☒	☐	Palaeontology
☐	☒	Animals and other organisms
☒	☐	Human research participants

Methods

n/a	Involved in the study	
☒	☐	ChIP-seq
☒	☐	Flow cytometry
☒	☐	MRI-based neuroimaging

Eukaryotic cell lines

Policy information about cell lines

Cell line source(s)	RAW264.7 macrophages were acquired from ATCC.
Authentication	Morphology checks, growth curve analysis, isoenzymology, and short tandem repeat profiling are all performed on this cell line by the supplier at the time of distribution.
Mycoplasma contamination	During all periods of maintenance RAW264.7 macrophages were regularly PCR-tested for mycoplasma contamination.
Commonly misidentified lines (See ICLAC register)	NA

Animals and other organisms

Policy information about studies involving animals; ARRIVE guidelines recommended for reporting animal research

Laboratory animals	Six- to ten-week-old female C57BL/6 mice were used in this study.
Wild animals	NA

Field-collected samples | NA
